# Risk and Prophylactic Management of Gallstone Disease in Bariatric Surgery: a Systematic Review and A Bayesian meta-analysis

**DOI:** 10.1007/s11605-022-05567-8

**Published:** 2023-01-10

**Authors:** Filipe Amorim-Cruz, Hugo Santos-Sousa, Miguel Ribeiro, Jorge Nogueiro, André Pereira, Fernando Resende, André Costa-Pinho, John Preto, Eduardo Lima-da-Costa, Bernardo Sousa-Pinto, C. R. I.-O. group

**Affiliations:** 1 Faculty of Medicine, University of Porto, Alameda Prof. Hernâni Monteiro, 4200-319 Porto, Portugal; 2 Obesity Integrated Responsibility Unit (CRI-O), São João University Medical Center, Alameda Prof. Hernâni Monteiro, 4200-319 Porto, Portugal; 3 Surgery Department, São João University Medical Center, Alameda Prof. Hernâni Monteiro, 4200-319 Porto, Portugal; 4 MEDCIDS - Department of Community Medicine, Information and Health Decision Sciences, Faculty of Medicine, Rua Dr. Plácido da Costa, 4200-450 Porto, Portugal; 5 CINTESIS - Center for Health Technologies and Services Research, University of Porto, Rua Dr. Plácido da Costa, 4200-450 Porto, Portugal

**Keywords:** Bariatric surgery, Symptomatic gallstone disease, Prophylactic cholecystectomy, Bayesian meta-analysis

## Abstract

**Background:**

The frequency and management of gallstone disease (GD) in bariatric patients, including the role of routine prophylactic concomitant cholecystectomy (CCY), are still a matter of debate. This study aims to assess the risk of de novo GD in patients undergoing bariatric surgery (BS) and their predictive factors, as well as mortality and morbidity in prophylactic CCY compared to BS alone.

**Methods:**

We performed a systematic review, searching PubMed, EMBASE, and Web of Science until April 2021. We performed a Bayesian meta-analysis to estimate the risk of GD development after BS and the morbidity and mortality associated with BS alone versus BS + prophylactic CCY. Sources of heterogeneity were explored by meta-regression analysis.

**Results:**

The risk of de novo post bariatric GD was 20.7% (95% credible interval [95% CrI] = 13.0–29.7%; *I*^2^ = 75.4%), and that of symptomatic GD was 8.2% ([95% CrI] = 5.9–11.1%; *I*^2^ = 66.9%). Pre-operative average BMI (OR = 1.04; 95% CrI = 0.92–1.17) and female patients’ proportion (OR = 1.00; 95% CrI = 0.98–1.04) were not associated with increased risk of symptomatic GD.

BS + prophylactic CCY was associated with a 97% probability of a higher number of postoperative major complications compared to BS alone (*OR* = 1.74, 95% CrI = 0.97–3.55; *I*^2^ = 56.5%). Mortality was not substantially different between the two approaches (*OR* = 0.79; 95% CrI = 0.03–3.02; *I*^2^ = 20.7%).

**Conclusion:**

The risk of de novo symptomatic GD after BS is not substantially high. Although mortality is similar between groups, odds of major postoperative complications were higher in patients submitted to BS + prophylactic CCY. It is still arguable if prophylactic CCY is a fitting approach for patients with a preoperative lithiasic gallbladder.

**Supplementary Information:**

The online version contains supplementary material available at 10.1007/s11605-022-05567-8.

## Introduction

Bariatric surgery (BS) has been identified as the most effective treatment for clinically severe obesity, resulting in sustained weight loss and significant improvement in obesity-related comorbidities.^1^ Despite its benefits, BS is associated with a 3–28% incidence of symptomatic gallstone disease (GD),^2^ which is five times higher than the healthy population^3^.

Understanding the risk factors associated with GD development may be crucial for risk stratification and distinct patient management, especially since GD risk factors in the general population may not be predictive in patients submitted to BS.^4, 5, 6^

The varying incidence of symptomatic GD after BS has resulted in controversies regarding whether prophylactic concomitant cholecystectomy (CCY) should be performed. Currently, there are three approaches on this subject: (i) routine prophylactic cholecystectomy concomitant to BS for all patients (BS + prophylactic CCY), (ii) a selective prophylactic CCY only for those with positive findings on pre-operative ultrasound, and (iii) medical prophylactic treatment with ursodeoxycholic acid (UDCA).^4,7,8^ There may be some arguments in favor or against each of these options—for example, CCY could avoid stone-related complications, including further hospitalization and surgery; however, it is a technically challenging procedure. On the other hand, cholecystectomy after BS, for symptomatic GD, is also a procedure associated with technical difficulties.^9,10^ Despite these controversies, evidence on all of these options has not been systematically assessed.

In this systematic review and meta-analysis, we aimed to (objective i) quantify the risk of de novo asymptomatic or symptomatic GD after BS, (objective ii) identify predictive factors associated with de novo GD after BS, and (objective iii) compare the morbidity and mortality of BS alone versus BS + prophylactic CCY.

## Material and Methods

This systematic review with meta-analysis follows the Preferred Reporting Items for Systematic Reviews and Meta-analyses (PRISMA) statement guidelines and the recommendations of the Cochrane Handbook for Systematic Reviews.^11,12^

### Eligibility Criteria

We included observational studies assessing BS as an obesity treatment for patients with a BMI ≥ 40 kg/m^2^ or BMI ≥ 35 kg/m^2^ with weight-related comorbidities. For objectives i and ii (risk of de novo post-bariatric GD and its predictive factors), the outcome to be reported was GD development. It was defined as de novo episodes of symptomatic biliary colic, acute cholecystitis, choledocholithiasis, cholangitis, and acute pancreatitis, or de novo asymptomatic evidence of cholelithiasis on post-operative ultrasound. For objective iii (comparison of the morbidity and mortality of BS alone versus BS + prophylactic CCY), any of the following outcomes needed to be reported: postoperative mortality, duration of surgery, hospital length-of-stay (LOS), and major postoperative complications. BS + prophylactic CCY was defined as cholecystectomy concomitant to bariatric surgery for asymptomatic patients (with no or asymptomatic gallstones confirmed by preoperative ultrasound), who had not been submitted to a previous cholecystectomy.

More detailed inclusion and exclusion criteria, for each specific objective, are shown in Supplementary Table 1.

### Search Strategy

We searched three electronic databases (PubMed, EMBASE, and Web of Science) from inception until April 6, 2021 (when our search was performed). Search queries are detailed in Supplementary Table 2. This search was supplemented by a gray literature search (conference papers, clinical trials—ongoing or unpublished), as well as hand-searching references of primary studies and other relevant reviews that were included. No restrictions were set regarding language or publication year.

### Study Selection and Data Collection Process

After removing duplicates, each study was independently assessed by two reviewers (F.C and M.R), first by title and abstract screening and then by full-text reading.

Two reviewers independently extracted data from selected studies using a predefined form purposely built for this systematic review. For each primary study, the following information was retrieved: authors’ identification, year of publication, country, study design, number of enrolled patients, type of performed BS, follow-up period, patients’ characteristics (distributions of gender, age, and pre-operative and post-operative body mass index (BMI)), frequency of co-morbidities, weight loss after surgery, and preoperative gallbladder status. The latter was classified as alithiasic or lithiasic gallbladder confirmed by ultrasonography. An alithiasic gallbladder was defined as a preoperative gallbladder without gallstones or sludge, and a lithiasic gallbladder was defined as a preoperative asymptomatic gallbladder with gallstones or sludge without being submitted to CCY.

For objectives i and ii (risk of de novo post-bariatric GD and its predictive factors), we also retrieved information on the number of patients: (i) at risk of GD, (ii) at risk of symptomatic GD only (with information retrieved also for the time to symptoms), (iii) who developed GD, (iv) who developed symptomatic GD only, (v) who developed each GD presentation (such as biliary colic, acute cholecystitis, choledocholithiasis, cholangitis, and acute pancreatitis), and (vi) undergoing postoperative cholecystectomy. Both patients with no symptoms of cholelithiasis and either preoperative negative gallstone findings or preoperative positive gallstone findings were at risk of de novo symptomatic GD. In contrast, only patients with preoperative negative gallstone findings and primarily asymptomatic were considered at risk for de novo asymptomatic GD. Whenever provided, we retrieved data separately based on preoperative gallbladder status. Data related to other biliary conditions, such as gallbladder carcinoma or polyps, were not retrieved.

For objective iii (comparison between BS alone versus BS + prophylactic CCY), the additional following information was concerned: (i) number of patients submitted to BS alone and BS + prophylactic CCY; (ii) reason for prophylactic CCY; (iii) surgery duration; (iv) LOS; (v) major postoperative complications (medical complications—cardiac arrest requiring cardiopulmonary resuscitation (CPR), respiratory failure, pneumonia, sepsis, venous thromboembolism, acute renal failure, and bleeding requiring transfusion; surgical complications—anastomotic leakage, organ space surgical site infection, the conversion rate of laparoscopic surgery, number of reoperations, and hospital readmission within 30 days); and (vii) postoperative mortality.

If distinct eligible publications reported data on the same patient cohort, the more recent and largest cohort was included. Authors were contacted whenever full texts were not available or to provide the relevant missing information. In study selection or data extraction, any disagreements between reviewers were resolved by consulting a third senior reviewer (H.S.S) to reach a final decision.

### Quality Assessment

The quality of primary studies was independently assessed by two reviewers (F.C and M.R) using the National Institutes of Health quality assessment criteria for observational studies.^13^ To reach a consensus, divergent opinions regarding quality assessment were discussed with a third reviewer (H.S.S). This tool consists of a form with 14 yes-or-no questions (related to the research question, study population, exposure, outcome, blinding, follow-up, and statistical analysis) and a final quality rating (good, fair, or poor), classifying the study according to its potential risk of bias.^13^ The question about assessors being blinded regarding the exposure status was not possible to assess in any of the included studies.

### Synthesis of Results

Given the inclusion of a large number of studies with no occurrence of events, we opted in performing a meta-analysis according to a Bayesian approach following a random-effects model based on a binomial likelihood.^14^ Compared to frequentist (“classical”) approaches, Bayesian meta-analysis deals more adequately with proportions equal to zero.

Bayesian methods provide estimations of posterior probability distributions of the parameters of interest, based on prior probability distributions and the observed data. In this study, for the risk of de novo post-bariatric GD, we computed the meta-analytical risk of GD and of symptomatic GD only. To compare outcomes between patients submitted to BS alone as index events versus those submitted to BS + prophylactic CCY, we computed meta-analytical odds ratio (OR) or mean differences (MD) depending on whether outcome variables were categorical or continuous, respectively. Of these results, we collected information on the mean values and respective 95% credible intervals (95% CrI; a range of values within which the true effect size measure lies, with a 95% probability).

Comparison between concomitant CCY and postoperative cholecystectomy was not quantitatively synthesized, due to the low number of available studies and substantial missing information.

Heterogeneity was assessed through an estimate of the *I*^2^ statistic—an *I*^2^ > 50% indicated substantial heterogeneity. Heterogeneity sources were explored through univariable meta-regression and subgroup analyses; – in particular, meta-regression allowed for the identification of potential predictive factors for the risk of de novo bariatric GD. Exponentials of the meta-regression coefficients were interpreted as OR. Finally, we also performed a separate meta-analysis for the development of symptomatic GD among patients with preoperative lithiasic versus alithiasic gallbladder.

For both the effect size measure and the τ parameter, we used uninformative prior distributions (dnorm (0, 0.00001) and dgamma (0.00001, 0.00001), respectively). We ran at least 40,000 iterations for each analysis with a burn‐in of 15,000 sample iterations. Meta‐analysis was performed using the rjags package of software R (version 3.5.0).

## Results

### Study Selection

**Fig. 1:**
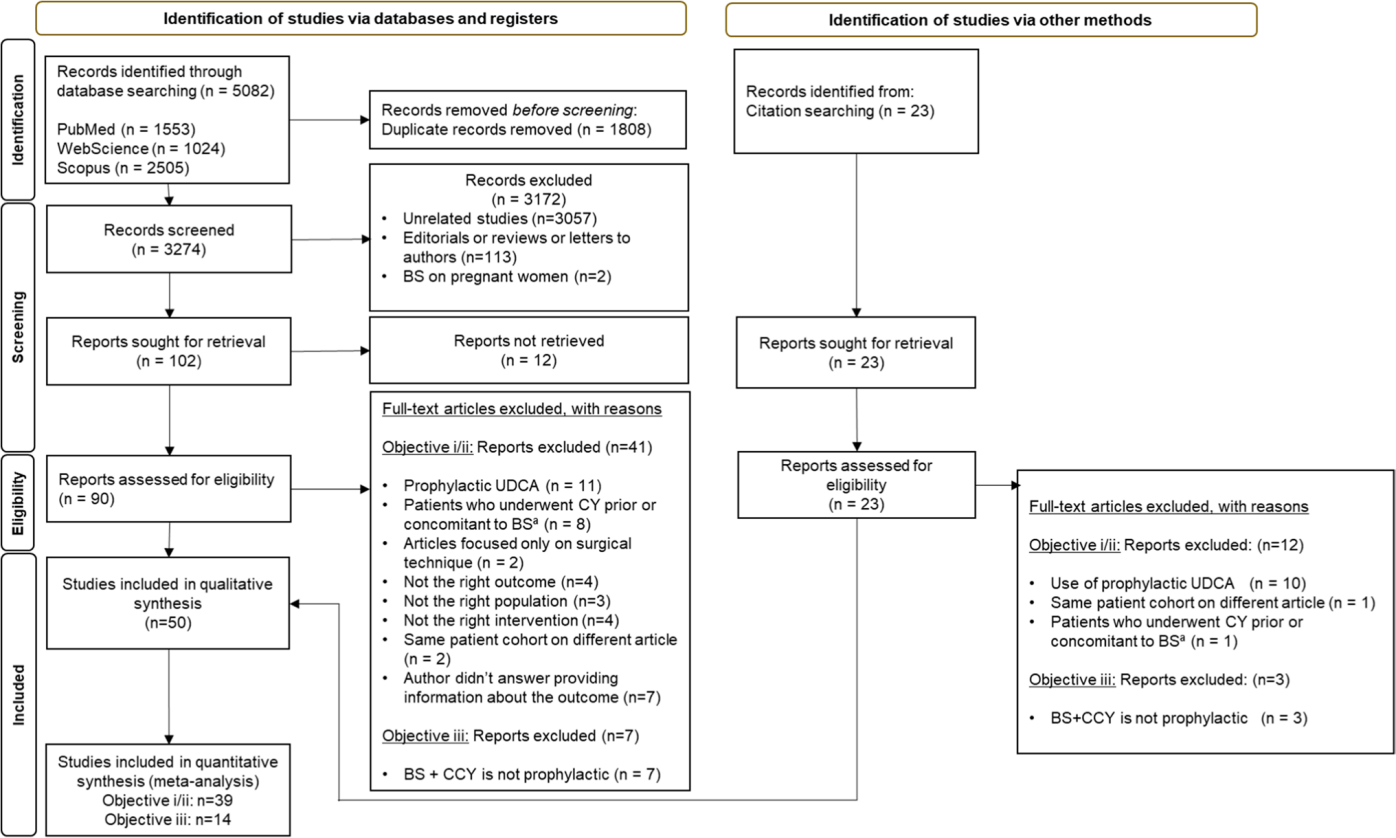
Flow diagram of study selection. BS, bariatric surgery; UDCA, Ursodeoxycholic acid; BS + CCY, prophylactic cholecystectomy concomitant to bariatric surgery; CY, cholecystectomy. ^a^This exclusion criteria is not applicable if patients with gallbladder in situ were individually reported. Objective i/ii—risk of de novo asymptomatic or symptomatic GD after BS and its predictive factors; objective iii—comparison of morbidity and mortality of BS alone versus BS + prophylactic CCY. 3 studies (Kim et al.; Tucker et al., Wanjura et al.) were used for 3 study objectives

The electronic literature search resulted in 5082 articles, of which 1808 were duplicates. After excluding 3184 records in the screening phase, 90 articles were fully read, of which a total of 42 were included in the systematic review (Fig. 1).^5,7,15–43,45–55^ Hand-searching resulted in 23 additional articles, of which 8 were included.^56–64^ Ten authors were asked for additional information, as outcomes of interest were missing. Seven did not answer, and their studies were excluded from objectives i and ii. In total, 50 articles were included—39 for answering objectives i and ii (assessment of the risk of de novo symptomatic or asymptomatic GD and its predictive factors)^5,7,15,17–24,26–37,39–46,48,49,51,52,55,56,58,62^ and 14 for objective iii (comparing BS alone versus BS + prophylactic CCY).^16,25,38,47,50–54,59–62,64^

### Study Characteristics

A summary of included studies is presented in Tables 1, 2, and 3. The remaining characteristics are reported in Supplementary Table 3.1–3.4.

**Table 1:** Studies general characteristics

Study	Year publication	Country	Study Design	BS	Preoperative gallbladder status		Quality rating^a^	
					Alithiasic gallbladder (*N*)	Lithiasic gallbladder (*N*)	G/F/P	Y/N
Abu Abeid Subhi	2002	Israel	PS	LAGB	134	0	P	7/5
Ahmed AR	2007	USA	RS	LRYGBP	400	0	G	8/4
Aldriweesh MA	2020	Saudi Arabia	RS	LSG (434) LAGB (56)	490	0	G	11/2
Alimogulları M	2020	Turkey	RS	LSG	111	0	G	10/3
Alsaif FA	2020	Saudi Arabia	RS	LSG	711	0	G	10/3
Amstutz S	2015	Switzerland	RS	LRYGBP	64	26	F	9/4
Anveden A	2020	Sweden	PS	OpenRYGBP (236) LAGB (1 519)	NR	NR	G	12/1
Aridi HD	2016	Lebanon	PS	LSG	NR	NR	G	13/0
Bastouly M	2009	Brazil	PS	Open RYGBP	20	0	P	7/4
Brockmeyer JR	2015	USA	RS	Open RYGBP (1 366) LSG (161)	1 111	1	F	7/6
Caruana JA	2005	USA	PS	RYGBP	401	98	F	10/3
Chen JH	2019	Taiwan	RS	Open RYGBP (1 155) LSG (1 156)	NR	NR	G	9/4
Coskun H	2014	Turkey	RS	LSG	32	16	F	9/3
Coupaye M	2015	France	PS	Open RYGBP (117) LSG (43)	160	0	G	11/2
Dakour Aridi HN	2017	Lebanon	RS	LSG	21 137	0	G	11/1
Dhondt M	2011	Belgium	PS	LRYGBP	521	104	G	12/1
de Oliveira CLB	2003	Brazil	RS	Open RYGBP	69	0	F	8/4
Dorman RB	2013	USA	RS	Open RYGBP (4 298) LRYGBP (28 648)	32 946	0	G	11/2
ElHadidi A	2019	Egypt	PS	LSG	755	95	G	11/2
Guzman HM	2019	Chile	RS	Open RYGBP (32) LSG (85) LAGB (59)	176	0	F	9/4
Hasan MY	2017	Singapore	RS	LSG	87	15	F	9/3
Juo YY	2018	USA	RS	Open RYGBP (300 919) LSG (205 315) LAGB (47 406)	536 904	16 755	G	12/1
Karadeniz M	2014	Turkey	RS	LRYGBP	46	0	F	8/4
Kim JJ	2009	USA	RS	Open RYGBP (264) LRYGBP (488)	273	298	G	10/3
Kiewiet RM	2006	Netherlands	RS	LAGB	103	0	G	9/3
Kızılkaya MC	2021	Turkey	RS	LSG	185	0	G	9/4
Lasnibat RJP	2017	Chile	RS	Open RYGBP (107) LAGB (114)	151	0	F	8/5
Li VKM	2009	USA	RS	LRYGBP (496) LSG (52)	548	0	F	10/3
Manatsathit W	2016	USA	RS	LSG	96	0	G	9/3
Melmer A	2015	Austria	PS	LSG (15) LAGB (94)	79	6	G	10/2
Moon RC	2014	USA	RS	LRYGBP (367) LSG (115) LAGB (104)	586	0	G	11/3
Morais M	2016	Portugal	RS	NR	581	72	G	12/1
Nagem R	2012	Brazil	PS	Open RYGBP	38	0	G	9/2
Nougou A	2008	Switzerland	RS	LRYGBP	632	82	F	8/4
OBrien PE	2003	Australia	PS	LAGB	809	0	F	7/5
Ostlund P	2012	Sweden	PS	LRYGBP (6 549) LAGB (6 894)	NR	NR	G	9/3
Santos BF	2014	USA	PS	Open RYGBP	32 041	1 034	F	8/1
Scott DJ	2003	USA	PS	LRYGBP	129	21	F	8/4
Sucandy I	2016	USA	RS	BPDwDS	239	63	F	10/3
Papavramidis S	2003	Greece	PS	NR	84	0	F	9/3
Patel JA	2009	USA	RS	LRYGBP	NR	NR	F	10/3
Patel KR	2009	USA	PS	LRYGBP	NR	NR	G	10/3
Pineda O	2017	Mexico	PS	NR	97	49	G	10/3
Portenier DD	2007	England	RS	LRYGBP	406	110	F	9/2
Sakcak I	2011	Turkey	PS	LAGB	137	0	F	10/3
Sioka E	2014	Greece	RS	LSG	106	32	G	11/2
Taha MIA	2006	Brazil	RS	LRYGBP	103	0	G	11/2
Tarantino I	2011	Switzerland	RS	LRYGBP	140	0	G	10/2
Tucker ON	2008	USA	RS	LRYGBP	1 462	82	F	7/5
Wanjura V	2018	Sweden	RS	LRYGBP	33 573	152	G	11/2
Wood SG	2019	USA	RS	LSG	4 048	0	G	11/2
Wood SG	2019	USA	RS	LRYGBP	2 820	0	G	11/2
Zilberstein B	2004	Brazil	PS	LAGB	263	17	F	8/4

*RS*, retrospective study; *PS*, prospective study; *BS*, bariatric surgery; *OpenRYGBP*, laparotomy Roux-en-Y gastric bypass; *LRYGBP*, laparoscopic Roux-en-Y gastric bypass; *LAGB*, laparoscopic adjustable gastric banding; *LSG*, laparoscopic sleeve gastrectomy; *BPDwDS*, biliopancreatic diversion with duodenal switch; *NR*, not reported

^a^NIH (National Institutes of Health) quality assessment criteria for observational studies—it is based on a quality rating of G (good), F (fair), and P (poor), and 14 questions of yes/no/not applicable/not reported/cannot determine. Y/N is the ratio of questions with positive answers (Y-yes) and negative answers (N–no)

Alithiasic gallbladder: a preoperative gallbladder without gallstones or sludge, confirmed by ultrasonography

Lithiasic gallbladder: a preoperative asymptomatic gallbladder with gallstones or sludge confirmed by ultrasonography, without being submitted to concomitant cholecystectomy

In included studies, Wood et al. provided separate data of laparoscopic sleeve gastrectomy (LSG) and laparoscopic Roux-en-Y Gastric Bypass (LRYGBP) with concomitant cholecystectomy (CCY), so we included these data as two individual studies

**Table 2:** Studies’ Clinical Characteristics for risk of de novo post-bariatric gallstone disease and its predictive factors

Study	Patients (*N*)	Age (mean ± SD, years)	Female (%)	Preoperative BMI (mean ± SD, kg/m^2^)	Follow-up (mon)	Asymptomatic + symptomatic de novo GD		Symptomatic de novo GD		Time to event (mon )	PosCY (N)
						Patients at risk (*N*)	Patients with GD (*N*)	Patients at risk (*N*)	Patients with GD (*N*)		
Abu Abeid Subhi	134	38.0	87.3	43.6	9.0	134	10	134	10	8.0	NR
Aldriweesh MA	490	36.8 ± 11.4	61.4	46.2 ± 6.9	27.0	490	32	490	32	48.0	NR
Alimogulları M	111	38.9	81.1	45.9 ± 6.1	20.6	111	41	111	13	13.8	41
Alsaif FA	711	34.6 ± 12.0	57.8	45.0 ± 10.3	12.0	NR	NR	711	25	12.4	NR
Amstutz S	64	NR	73.4	43.8	44.0	64	33	64	22	25.43	16
Anveden A	1 755	47.2 ± 5.9	32.0	42.4 ± 4.5	254.4	NR	NR	1755	307	NR	230
Aridi HD	319	30.2 ± 7.4	61.4	42.9 ± 7.1	24.0	NR	NR	319	24	14.0	24
Bastouly M	20	39.3	80.0	46.1 ± 5.2	35.0	20	13	20	6	6.0	0
Brockmeyer JR	1 527	44.6	NR	47.7	140.0	NR	NR	1111	91	20.4	91
Chen JH	2 317	32.8 ± 8.6	57.4	NR	71.4	NR	NR	2317	67	33.89	NR
Coupaye M	160	41.7 ± 11.1	90.6	44.9 ± 6.0	24.0	160	52	160	20	14.96	23
de Oliveira CLB	103	39.0	NR	54.1	12.0	36	19	36	19	12.0	NR
Dhondt M	625	38.1	69.0	41.5	51.0	521	34	625	43	17.4	39
ElHadidi A	850	35.3 ± 7.7	70.8	39.5 ± 1.0	19.83	755	218	850	235	11.67	NR
Guzman HM	176	37.8 ± 10.5	54.5	37.5	12.0	176	65	176	NR	NR	NR
Hasan MY	102	43.0	58.8	41.7 ± 6.5	28.4	NR	24	101	1	NR	1
Karadeniz M	46	39.1 ± 10.0	89.1	47.8 ± 7.0	28.6	46	10	46	6	18.0	5
Kim, Jin-Jo	454	42.6 ± 9.3	NR	54.2 ± 10.4	30.6	273	9	273	9	14.7	NR
Kiewiet RM	103	42.3 ± 9.1	90.3	44.2 ± 5.6	56.1	103	31	103	7	37.0	7
Kızılkaya MC	185	36.4 ± 9.5	81.6	44.2 ± 5.0	6.0	185	27	185	3	NR	NR
Lasnibat RJP	221	44.9	NR	39.7 ± 39.7	12.0	141	6	141	1	0.0	6
Li VKM	548	42.7 ± 74.8	74.8	48.0	26.1	NR	NR	548	45	10.11	NR
Manatsathit W	96	44.5 ± 12.0	79.2	49.1 ± 7.9	14.3	96	48	96	22	353.17	17
Melmer A	109	55.3 ± 10.5	NR	NR	125.5	79	19	79	12	21.6	12
Moon RC	586	43.4 ± 11.7	76.1	45.9 ± 7.2	14.9	NR	NR	586	28	12.64	28
Morais M	653	42.0 ± 10.6	85.0	44.8 ± 5.4	26.0	NR	NR	653	24	16.5	9
Nagem R	38	41.7	89.5	48.1	36.0	38	11	38	6	NR	11
O'Brien PE	809	NR	NR	NR	42.0	NR	NR	809	55	NR	55
Ostlund M	13 443	40.0	74.4	NR	88.1	NR	NR	13,443	722	15.0	1,149
Papavramidis S	84	33.7 ± 9.2	61.9	52.6 ± 10.0	24.0	NR	NR	84	34	16.0	34
Patel JA	1 050	43.4	75.2	49.3	32.3	NR	NR	1050	52	10.9	52
Patel KR	199	43.0	85.9	50.1	14.7	NR	NR	199	12	9.5	12
Pineda O	146	38.5	80.8	NR	24.0	97	31	146	5	NR	5
Portenier DD	1 057	NR	NR	49.0	30.0	903	66	984	80	11.2	80
Sakcak I	137	29.6 ± 6.1	78.8	46.8 ± 6.6	23.8	NR	NR	137	7	7.4	7
Sioka E	150	NR	NR	46.1	26.0	106	8	138	8	NR	7
Taha MIA	103	NR	82.5	48.6 ± 7.2	12.0	103	48	103	22	6.2	NR
Tucker ON	1 544	NR	NR	NR	30.5	NR	NR	1544	104	18.2	85
Wanjura V	33 725	40.7 ± 11.0	78.3	42.5 ± 5.4	24.0	33,573	1133	33,573	1,133	11.8	1,133

*BMI*, body index mass (kg/m2); *GD*, gallstone disease; *PosCY*, postoperative cholecystectomy; *SD*, standard deviation; *mon*, months; *NR*, not reported

**Table 3:** Studies’ clinical characteristics for comparison of the morbidity and mortality of bariatric surgery alone versus prophylactic cholecystectomy concomitant to bariatric surgery

Study	Patients (*N*)	Age (mean ± SD, years)	Female (%)	Preoperative BMI (mean ± SD, kg/m^2^)	Follow-up (days)	BS + CCY (*N*)	BS alone (*N*)	Reason for BS + CCY	
								US − (*N*)	US + (*N*)
Ahmed AR	400	43.0 ± 18.5	84.8	NR	30	200	200	200	0
Coskun H	48	35.5 ± 10.7	72.9	51.0 ± 5.47	3	16	32	0	16
Dakour-Aridi HN	21 137	44.8 ± 11.3	78.2	46.3 ± 8.1	30	422	20,715	422	0
Dorman RB	32 946	NR	79.3	NR	30	1731	31,215	1731	0
Juo, Yen-Yi	553 659	NR	78.1	NR	182	18,268	535,391	5 261	13,007
Kim, Jin-Jo	752	42.6 ± 9.3	79.8	54.2 ± 10.4	949	298	273	0	298
Nougou A	772	39.8	75.9	45.6	NR	655	59	576	79
Santos BF	33 075	44.0 ± 11.0	NR	47.0 ± 8.0	3	1034	32,041	0	1034
Sucandy I	361	44.8 ± 10.1	73.1	50.5 ± 20.9	961	63	239	0	63
Tarantino I	274	41.8 ± 27.2	73.7	46.9 ± 6.1	986	134	140	34	100
Tucker ON	1 669	NR	NR	NR	946	123	1464	0	122
Wanjura V	33 725	40.7 ± 11.0	78.3	42.5 ± 5.4	730	67	33,573	0	67
Wood SG	4 048	45.3 ± 12.0	84.7	44.9 ± 8.1	30	2 024	2024	2024	0
Wood SG	2 820	45.6 ± 11.9	80.2	46.3 ± 8.7	30	1410	1410	1410	0
Zilberstein B	308	36.4 ± 23.6	72.4	41.6	558	17	290	0	17
Study	Operative time (mean ± SD, minutes)			Hospital of stay (mean ± SD, days)		Postoperative complications		Postoperative mortality (*N*)	
	BS + CCY	BS alone	Difference	BS + CCY	BS alone	Total *N* (%)		BS + CCY	BS alone
						BS + CCY	BS alone		
Ahmed AR	125.0 ± 28.0	96.0 ± 23.0	29.0	2.0 ± 0.2	2.0 ± 0.3	17 (8.5)	17 (8.5)	0	0
Coskun H	157.2 ± 40.0	95.7 ± 26.2	61.5	3.6 ± 0.9	3.4 ± 0.8	2 (6.3)	3 (18.8)	0	0
Dakour-Aridi HN	128.2 ± 53.9	95.3 ± 47.3	32.9	2.1 ± 6.0	2.3 ± 4.4	24 (5.7)	820 (4.0)	19	1
Dorman RB	NR	NR	NR	NR	NR	114 (6.6)	1 528 (5.0)	60	6
Juo, Yen-Yi	NR	NR	NR	2 ± 1	2.0 ± 1.0	1 115 (6.2)	20 345 (3.8)	NR	NR
Kim, Jin-Jo	198.4 ± 61.9	177.7 ± 57.7	20.7	3.3 ± 5.5	2.9 ± 6.1	NR	NR	5	3
Nougou A	142.6	158.5	− 15.6	4.0	4.0	1 (0.002)	0	NR	NR
Santos BF	NR	NR	NR	2.6	2.5	60 (5.8)	1615 (5.0)	51	3
Sucandy I	302.8 ± 62.8	290.8 ± 67.8	12.0	3.0	3.5	NR	NR	0	0
Tarantino I	220.7 ± 58.4	234.8 ± 70.7	− 14.1	7.3 ± 4.7	10.8 ± 6.0	NR	NR	NR	NR
Tucker ON	NR	NR	NR	2.8	2.6	4 (3.2)	0	0	0
Wanjura V	145.0 ± 53.0	74⋅2 ± 36.3	70.8	NR	NR	26 (38.8)	2 865 (8.5)	NR	NR
Wood SG	103.7 ± 46.2	76.7 ± 40.6	27.0	1.9 ± 1.8	1.8 ± 3.0	69 (3.4)	51 (2.5)	2	0
Wood SG	149.6 ± 60.8	121.9 ± 59.8	27.7	2.4 ± 3.3	2.2 ± 2.7	100 (7.1)	84 (6.0)	2	0
Zilberstein B	86.0 ± 17.0	58.0 ± 19.0	28.0	0.6 ± 0.1	0.7 ± 0.1	0	14 (4.8)	0	0

*BMI*, body mass index (kg/m^2^); *SD*, standard deviation; *US − *, preoperative negative ultrasonography findings for gallstone disease; *US* + , preoperative positive ultrasonography findings for gallstone disease; *BS*, bariatric surgery; *BS* + *CCY*, prophylactic cholecystectomy concomitant to bariatric surgery; *NR*, not reported

For objectives i and ii, included studies were published between 2004 and 2019, with a cumulative sample size of 64,950 patients. Most were retrospective cohort studies (*n* = 24, 61.5%). The mean participants’ age was 40.9 years (*SD* = 9.3 years) with a female predominance (74.0%). Laparoscopic Roux-en-Y gastric bypass (LRYGBP) was the most-performed BS type (13 studies, *n* = 46, 313, 71.3%). The mean follow-up period was of 29.0 months.

For objective iii, included studies were published between 1995 and 2006, with a cumulative sample size of 685,994 patients. Open Roux-en-Y gastric bypass (RYGBP) was the most-performed BS type (5 studies, *n* = 653 405, 95.2%). Among patients undergoing prophylactic CCY (*n* = 26 461, 4%), 44% had negative findings for GD in pre-operative ultrasonography.

### Risk of Developing *De Novo* Gallstone Disease

A total of 38,210 asymptomatic patients with only preoperative negative GD findings were at risk of de novo postoperative GD. The Bayesian meta‐analysis identified a post-bariatric risk of de novo GD of 20.7% (95% credible interval [95% CrI] = 13.0–29.7%), even though with severe heterogeneity (*I*^2^ = 75.4%). A higher BMI was associated, – with a 96% probability, – with higher odds of de novo GD (*OR* = 1.11; 95% CrI = 0.99–1.22). Other results of univariable meta-regression are presented in Supplementary Table 4.

A total of 63 938 asymptomatic patients with either preoperative negative or positive gallstone findings were at risk of de novo symptomatic GD. A total of 3312 developed symptomatic GD, corresponding to a meta-analytical risk of 8.2% (95% CrI = 5.9–11.1%), even though with high heterogeneity (*I*^2^ = 66.9%, Table 4). The three most common clinical presentations were biliary colic (*n* = 1559, 65.5%), cholecystitis (*n* = 120, 14.7%), and symptomatic choledocholithiasis (*n* = 37, 4.5%) (Supplementary Table 3.1). Almost all patients required postoperative cholecystectomy (*n* = 3 179, 96%). The results of univariable meta-regression and subgroup analyses are presented in Table 4. Regarding studies’ methodological characteristics, retrospective studies were associated, – with a 94% probability,—with lower odds of de novo symptomatic GD (*OR* = 0.60; 95% CrI = 0.31–1.10).

**Table 4:** Results of metaregression and subgroup analyses for the risk of de novo post-bariatric symptomatic gallstone disease after bariatric surgery

	Number of studies	Number of patients	Subgroup analyses		Univariable metaregression—OR (95% CrI) [% iterations with OR < 1]
			Percent of risk of de novo symptomatic GD (95%CrI)	*I*^2^	
All	39	63 938	8.2 (5.9–11.1)	66.9	
Year of publication	39	63 938	a	a	0.99 (0.99–1.00) [99%]
Study design					
Prospective design	15	18 798	11.2 (6.9–16.7)	61.3	b
Retrospective design	23	45 140	6.6 (4.1–10.4)	70.7	0.60 (0.31–1.10) [94%]
Average of Age	27	58 656	a	a	0.99 (0.92–1.06) [59%]
Female Patients’ Proportion	29	58 823	a	a	1.00 (0.98–1.04) [37%]
Pre-operative Average BMI	26	42 917	a	a	1.04 (0.92–1.17) [26%]
Follow-up	31	61 176	a	a	1.00 (1.00–1.01) [25%]
Preoperative gallbladder status					
Lithiasic gallbladder	7	37 492	6.1 (1.6–14.2)	76	b
Alithiasic gallbladder	22	4 601	10.2 (6.0–15.6)	70.3	2.24 (0.72–5.23) [9%]
Type of BS					
No RYGBP subgroup	25	57 726	7.7 (5.6–10.3)	56.9	b
RYGBP subgroup	10	6 212	8.2 (2.5–22.0)	82.7	1.10 (0.47–2.52) [40%]
No LRYGBP subgroup	23	10 900	8.3 (5.0–12.5)	69.1	b
LRYGBP subgroup	13	53 038	7.5 (4.8–11.7)	60.7	0.93 (0.47–1.87) [59%]
No LSG subgroup	21	55 960	8.8 (5.4–13.1)	68	b
LSG subgroup	15	7 978	7.1 (4.4–13.0)	64.4	0.84 (0.40–1.67) [69%]
No LAGB subgroup	25	46 085	9.1 (5.8–13.2)	69	b
LAGB subgroup	10	17 853	6.5 (3.8–10.6)	52.6	0.69 (0.32–1.44) [82%]
Quality rating					
Poor/fair quality	16	40 558	9.6 (4.5–16.9)	77	b
High quality	22	23 380	7.5 (5.2–10.6)	59.1	0.60 (0.30–1.14) [94%]

*BMI*, body mass index (kg/m2); *OpenRYGBP*, laparotomy Roux-en-Y gastric bypass; *LRYGBP*, laparoscopic Roux-en-Y gastric bypass; *LAGB*, laparoscopic adjustable gastric banding; *LSG*, laparoscopic sleeve gastrectomy; *GD*, gallstone disease; *OR*, odds ratio; *CrI*, credible intervals

^a^No subgroup analysis was performed, as this is a continuous variable (we were only able to perform meta-regression analysis)

^b^Reference category

Pre-operative average BMI (*OR* = 1.04; 95% CrI = 0.92–1.17) and female patients’ proportion (*OR* = 1.00; 95% CrI = 0.98–1.04) did not impact the risk of de novo symptomatic GD. Although none of the assessed bariatric surgery types had a strong impact on GD risk, laparoscopic gastric banding (LAGB) was associated, – with an 82% probability,—with lower odds of de novo symptomatic GD. Insufficient data on co-morbidities and weight loss after surgery did not allow meta-regression analysis models to include these variables.

### Comparison between bariatric surgery alone versus prophylactic cholecystectomy concomitant to bariatric surgery

Bayesian meta‐analysis identified that postoperative mortality was not substantially different between BS alone versus BS + prophylactic CCY (*OR* = 0.79; 95% CrI = 0.03–3.02; *I*^2^ = 20.7%). BS + prophylactic CCY was associated with 97% probability of a higher number of postoperative major complications compared to BS alone (*OR* = 1.74, 95% CrI = 0.97–3.55; *I*^2^ = 56.5%). The odds of organ space surgical site infection were similar between groups (*OR* = 0.97, 95% CrI = 0.05–4.71; *I*^2^ = 52.8%) (Table 5). Insufficient data on some complications, namely the conversion rate of laparoscopic surgery and hospital readmissions within 30 days did not allow such outcomes to be analyzed through meta-analysis.

**Table 5:** Outcomes measured in the comparison between bariatric surgery alone versus prophylactic cholecystectomy concomitant to bariatric surgery

	Number of studies	Number of patients	OR (95% CrI)	*I*^*2*^	MD (95% CrI)	*I*^*2*^
Total of major complications	13	684 607	1.74 (0.97–3.55)	56.5%		
Pneumonia	4	60 951	1.26 (0.23–5.34)	58.4%		
Venous thromboembolism	3	39 814	0.34 (0.04–1.40)	25.3%		
Bleeding transfusion	7	61 351	1.05 (0.28–2.09)	33.5%		
Organ space surgical site infection	5	60 951	0.97 (0.05–4.71)	52.8%		
Postoperative mortality	11	97 564	0.79 (0.03–3.02)	20.7%		
Surgery duration	13	619 323			29.2 (17.9–40.7)	89.3%
Hospital length-of-stay	11	64 645			− 0.1 (− 1.0–0.5)	74.3%

*OR*, odds ratio; *MD*, mean differences; *CrI*, credible intervals

Univariable meta-regression results are presented in Table 6. Neither age (*OR* = 1.22, 95% CrI = 0.87–1.68) nor female patients’ proportion (*OR* = 0.95, 95% CrI = 0.87–1.04) were associated with a relevant impact on the association between BS alone versus BS + prophylactic CCY on the occurrence of postoperative major complications. Although any type of bariatric surgery had no strong impact on postoperative major complications, they slightly changed through bariatric procedures—laparoscopic gastric banding (LAGB) was the one associated with lower chances (87%) of postoperative major complications between BS + prophylactic CCY versus BS alone (*OR* = 0.42, 95% CrI = 0.04–1.97). Moreover, prophylactic cholecystectomy concomitant to laparoscopic sleeve gastrectomy (LSG), compared to prophylactic cholecystectomy concomitant laparoscopic Roux-en-Y gastric bypass (LRYGBP) (*OR* = 0.66, 95% CrI = 0.16–2.59 versus *OR* = 2.33, 95% CrI = 0.28–10.27), had a tendentially lower probability of postoperative major complications. Regarding Roux-en-Y gastric bypass, comparing open and laparoscopic approaches, the probability of postoperative major complications was not too dissimilar (*OR* = 1.64, 95% CrI = 0.33–4.80 versus *OR* = 2.33, 95% CrI = 0.28–10.27).

**Table 6:** Results of metaregression for postoperative major complications between bariatric surgery alone versus prophylactic cholecystectomy concomitant to bariatric surgery

	Number of studies	Number of patients	Univariable metaregression—OR (95% CrI) [% iterations with OR < 1]
Year of publication	13	684 607	1.00 (1.00–1.00) [62%]
Study design (retrospective)	13	651 224	3.77 (0.51 − 5.945) [9%]
Mean age	9	96 333	1.22 (0.87 − 1.68) [25%]
Female patients’ proportion	10	649 863	0.95 (0.87 − 1.04) [80%]
Preoperative average BMI	8	95 933	0.80 (0.68 − 0.98) [99%]
Follow-up	11	683 835	1.00 (1.00 − 1.00) [0%]
OpenRYGBP	5	653 405	1.64 (0.33 − 4.80) [36%]
LRYGBP	7	38 607	2.33 (0.28 − 10.27) [33%]
LSG	4	578 892	0.66 (0.16 − 2.59) [74%]
LAGB	2	553 967	0.42 (0.04 − 1.97) [87%]
Quality rating	13	615 010	0.49 (0.16 − 1.27) [93%]

*OR*, odds ratio; *MD*, mean differences; *CrI*, credible intervals

*BMI*, body mass index (kg/m2); *OpenRYGBP*, laparotomy Roux-en-Y gastric bypass; *LRYGBP*, laparoscopic Roux-en-Y gastric bypass; *LAGB*, laparoscopic adjustable gastric banding; *LSG*, laparoscopic sleeve gastrectomy; *OR*, odds ratio; *CrI*, credible intervals

Patients submitted to BS + prophylactic CCY had a longer operative time—more than 29.2 min (95% CrI = 17.9–40.7), even though there was severe heterogeneity found (*I*^2^ = 89.3%). There were no relevant differences in hospital LOS (MD =  − 0.1 days; 95% CrI =  − 1.0–0.5; *I*^2^ = 74.3%) (Table 5).

### Risk of Bias of Individual Studies

The results of the risk of bias assessments for included primary studies are presented in Table 1, and a detailed description is reported in Supplementary Table 5.1 and Table 5.2. Most studies (*n* = 47, 94%) did not justify the sample size, and 39 studies (78%) did not adjust for any potential confounding variables. For the remaining parameters, most studies were associated with a low risk of bias. Bastouly et al.^56^ were considered to have a high risk of bias, namely selection bias since patients were selectively invited.

In fact, when considering only high-quality studies, the risk of symptomatic GD is of 7.5% (95% CrI = 5.2–10.6%; *I*^2^ = 59.1%). Such a trend was not observed for asymptomatic or symptomatic GD. Having a high risk of bias was also related,—with 93% probability,—with a weak association between BS alone versus BS + prophylactic CCY on the occurrence of postoperative major complications (OR 0.49; 95% CrI = 0.16–1.27).

## Discussion

The main findings of our study were the following: (1) the risk of developing de novo symptomatic gallstone disease after BS is not substantially high (8.2%), although three times higher than the healthy population; (2) GD predictive factors after BS are not similar to those of the general population, except for preoperative average of BMI in asymptomatic or symptomatic GD; and (3) patients who underwent prophylactic CCY had a longer operative time and a higher rate of postoperative complications than those who underwent BS alone, but mortality and hospital LOS were similar.

Some of the pathogenic mechanisms that can explain why patients after BS are at risk of developing GD include an increased biliary cholesterol concentration following rapid weight loss, gallbladder hypomotility secondary to vagal nerve resection and a decreased cholecystokinin secretion, an increased secretion of calcium and biliary mucin, and a disturbed enterohepatic circulation of biliary salts.^5,6,37,65^ Our meta-analysis showed that the risk of developing symptomatic GD is 8.2%, in a mean follow-up of 29.0 months. Warschkow et al.^8^ reported a similar percentage (6.8%), although the cumulative sample size was lower and only LRYGBP was performed. Our risk might be slightly underestimated since a clear decrease in the risk of de novo symptomatic GD was observed for retrospective studies, which might be explained by information bias. In fact, considering only prospective studies, this risk rises to 11.2%. Therefore, our findings, regarding the risk of developing symptomatic GD after BS, may call into question the pertinence of prophylactic CCY. Theoretically, one of the reasons to routinely perform cholecystectomy concomitant to BS concerns the prevention of later biliary complications (symptomatic choledocholithiasis, acute cholangitis, and biliary pancreatitis), mainly because endoscopic retrograde cholangiopancreatography (ERCP) is routinely impossible to perform after LRYGBP.^8,66^ In the present study, similar to other meta-analyses,^8,67^ symptomatic choledocholithiasis occurred in 37 patients (4.5%) and acute pancreatitis in 23 (2.8%). As a rare event, it does not uphold a prophylactic CCY.

Understanding predictive factors for gallstone formation after BS could influence distinct patient management, including a selective approach for BS + prophylactic CCY. To the best of our knowledge, this is the first meta-analysis exploring this topic. Through meta-regression and subgroup analyses, we found that neither higher pre-operative BMI, nor female patients’ proportion appear to be risk factors. However, we were only able to assess data at the study level. Assessing whether having a high BMI or being a female as risk factors for GD development would require an assessment of individual participant data. In our study, LAGB was associated with lower odds of de novo symptomatic GD. This could be explained by the fact that this restrictive bariatric procedure does not alter gastrointestinal transit, biliary contraction mechanisms, and enterohepatic circulation.^36,68^ On the contrary, in the RYGBP procedure, the altered anatomy, the division of the vagus nerve, and reduced cholecystokinin may lead to gallbladder dysmotility.^18,69^ It was not possible to understand the association between symptomatic GD and excessive weight loss. Although rapid weight loss is classically pointed to as the main predictive factor,^7^ it is not consensual across individual studies.^4,6,26,27,36^

We performed a separate meta-analysis to understand the impact of preoperative gallbladder status (lithiasic versus alithiasic) in the development of symptomatic GD. There is a 30% probability of symptomatic GD being more common in patients with preoperative lithiasic gallbladder than with preoperative alithiasic gallbladder (*OR* = 1.51; 95% CrI = 0.31–3.77). Moreover, according to the literature, severe biliary complications after bariatric surgery (symptomatic choledocholithiasis, acute cholangitis, and biliary pancreatitis) are more common in patients with asymptomatic preoperative gallstones.^70,71^ These arguments might reinforce a more selective approach, where patients with asymptomatic GD would undergo prophylactic CCY, given that, after RYGBP, ERCP is impossible to perform. However, from an expectant management perspective, surgery would be avoided in 88% of these patients and, consequently morbidity of a concomitant procedure. Furthermore, as ultrasound has a low sensibility in patients with obesity, there is no ideal screening method for patient selection. In meta-regression, studies enrolling only patients with alithiasic gallbladders were associated with higher odds of de novo symptomatic GD compared to studies enrolling patients with alithiasic and lithiasic gallbladders. These two approaches (separate meta-analysis versus meta-regression) may seem to contradict each other. However, the first approach evaluates individual participant data, whereas the second evaluates data at a study level, aiming to measure covariable impact in heterogeneity.

Regarding safety, we observed similar results in postoperative mortality and hospital LOS in patients submitted to BS + prophylactic CCY in comparison to BS alone. On the other hand, operative time and odds of postoperative major complications were higher in patients submitted to BS + prophylactic CCY. Concomitant cholecystectomy is a technically challenging procedure, which could account for these findings. Gallbladder position, often embedded in a steatosis liver, the inadequate position of the trocars, and operator fatigue are some issues to be pointed out.^4,66,72^ It is worth mentioning that there could be a selection bias present, when assessing the outcome operation BS + prophylactic CCY. In some studies,^38,51,62^ patients had an indication for prophylactic CCY, but the procedure was abandoned due to insufficient exposure of the right upper quadrant, patients’ comorbidities, surgeon preference, or technical difficulties. Other meta-analyses reached identical results,^10,67,70,73^ but it is worth noting that these authors studied cholecystectomy concomitant to bariatric surgery for both prophylactic and symptomatic management.

Our results should be interpreted with caution, owing to the observed heterogeneity, which suggests important differences between studies.

Through meta-regression, covariates such as age and the proportion of females did not have a relevant impact on the association between BS alone versus BS + prophylactic CCY in concern to postoperative major complications. We also found that no type of bariatric surgery had a strong impact on postoperative major complications. In the open RYGBP era, prophylactic concomitant cholecystectomy was advocated due to the higher risk of symptomatic gallstone disease, technical difficulties in re-operation, and low morbidity with concomitant cholecystectomy. With current minimally invasive procedures, postoperative major complications seem to outdo the relatively low incidence of symptomatic gallstone.^7,51^ There are only a few studies and no systematic reviews comparing postoperative complications between LSG and LRYGBP with prophylactic CCY. Based on our data, the addition of prophylactic CCY either to LSG or LRYGBP was not associated with an increase in major complications.

Postoperative cholecystectomy safety could modify the perspective regarding prophylactic CY. While it would have been interesting to explore the morbidity and mortality of BS + prophylactic CCY versus postoperative cholecystectomy, we were not able to perform a meta-analysis due to a limited number of studies. Reduced intra-abdominal fat and liver size after BS^26^ make delayed cholecystectomy technically easier to perform. Warschkow et al.^8^ found that the risk of suffering a complication during subsequent cholecystectomy is only 0.1%. Randomized controlled studies are still needed to assess surgical complications, operative time, LOS, and mortality associated with subsequent cholecystectomy.

This systematic review has some limitations worth noting. First, severe heterogeneity was found, explained by different study designs and eligibility criteria. To explore possible sources of heterogeneity, meta-regression and subgroup analysis were preformed, even though they did not account for all heterogeneity. The impact of distinct exclusion criteria, mainly in objectives i and ii (risk of de novo post-bariatric GD and its predictive factors), might be an explanation—some authors^18,32,58^ excluded patients with preoperative positive findings for GD in ultrasonography, while others^20,23,29^ did not, leading to a possible overestimation of GD development. Second, this systematic review lacks randomized controlled trials to provide strong evidence to support our findings. Third, almost half of the included studies did not have a low risk of bias, which could impact our results on the risk of de novo GD and postoperative complications rate, particularly, as the quality of the primary studies’ was found to be a moderator variable of heterogeneity.

There are also strengths in our study. The main methodological strength of this study is its meta-analytical approach to quantitative synthesis. The main advantage of Bayesian meta‐analysis is its use of exact methods, dealing more adequately with zero‐cells. Second, we performed a comprehensive search, encompassing three different electronic bibliographic databases and not using exclusion criteria based on the date or language of publication. Third, regarding eligibility criteria, to estimate the risk of post-bariatric de novo GD, studies that included prophylactic treatment with UDCA after BS or patients submitted to cholecystectomy prior or concomitant to BS, were excluded avoiding the risk of underestimation. Finally, meta-regression and subgroup analysis allowed the identification of predictive factors for gallstone formation.

In conclusion, after BS, the risk of developing GD is not substantially high, and severe biliary complications are extremely rare. Although there were no substantial differences in postoperative mortality or hospital length-of-stay, the determined risk of symptomatic GD and the higher risk of postoperative complications do not seem to justify performing prophylactic CCY in patients with alithiasic gallbladder. Doubts remain if a selective approach is advantageous since patients with preoperative gallbladder pathology have some increased risk of symptomatic GD. Randomized controlled studies might be considered to further clarify the role of prophylactic CCY as a selective approach. For future studies, we make the following recommendations: (i) postoperative cholecystectomy versus prophylactic CCY safety should be further explored; (ii) excess weight loss should be reported more consistently since findings are still not consensual regarding its lithogenic influence.

## Supplementary Information

Below is the link to the electronic supplementary material.

Supplementary file1 (DOCX 121 KB)

## Data Availability

The data underlying this article will be shared on reasonable request to the corresponding author.
