# Supplementary material for: Risk and Prophylactic Management of Gallstone Disease in Bariatric Surgery: a Systematic Review and A Bayesian meta-analysis
Source: J Gastrointest Surg. 2023 Jan 10;27(2):433–48. doi: 10.1007/s11605-022-05567-8 (PMC9974690; doi:10.1007/s11605-022-05567-8)
Supplement: Supplementary file 1 — Supplementary file1 (DOCX 121 KB) [file 11605_2022_5567_MOESM1_ESM.docx]

**Supplementary Material**

**Supplementary Table 1** Detailed Inclusion and Exclusion for studies selection

| **Inclusion criteria** | **Exclusion Criteria** |
| --- | --- |
| For both parts |  |
| (i) Indication for bariatric surgery was obesity treatment | (i) Reviews, Case reports, Editorials, Letters to authors, Ecological studies |
| (ii) Adults (age ≥ 18 years) | (ii) BS on pregnant women or elderly |
| (iii) Patients with a BMI ≥ 40 kg/m2 or BMI ≥ 35 kg/m2 with weight-related comorbidities. | (iii) Studies in which data could not be extracted from the pooled results |
|  | (iv) Studies focus solely on description of BS complications or Surgical Technique |
|  | (v) Use of prophylactic UDCA treatment after BS |
| For part I |  |
| (i) Studies reporting the number of patients with *de novo* symptomatic or asymptomatic GD; and with symptomatic GD only | (i) Studies whom patients were submitted to a previous or concomitant cholecystectomy and data analysis was not made separately ᵃ |
| (ii) Follow up more than 3 months after BS | (ii) Studies stating secondary or revisional bariatric surgery |
|  | (iii) Studies only reporting the number of patients submitted to postoperative cholecystectomy |
| For part II |  |
| (i) Comparing BS + prophylactic CCY versus only BS | (i) Studies with other surgical procedures done during prophylactic CCY |
| (ii) Studies stating one of the following complications: risk of mortality, duration of surgery, LOS, major complications |  |

BS: Bariatric Surgery; UDCA: Ursodeoxycholic acid; GD: gallstone disease; BS+CCY: prophylactic cholecystectomy concomitant to bariatric surgery; LOS: hospital length of stay

ᵃ This exclusion criteria is not applicable if patients with gallbladder in situ were individually reported

**Supplementary Table 2** – Search terms and queries

| MEDLINE/PubMed | (("bariatric Surgery"[All Fields] OR "bariatric Surgery"[MeSH Terms] OR "Bariatric"[All Fields] OR "Metabolic Surgery"[All Fields] OR "obesity surgery"[All Fields] OR "Gastric Bypass"[All Fields] OR "Gastric Bypass"[MeSH Terms] OR "Roux-en-Y Gastric Bypass"[All Fields] OR "gastric band*"[All Fields] OR "Vertical Banded Gastroplasty"[All Fields] OR "Gastric Sleeve"[All Fields] OR "Sleeve Gastrectomy"[All Fields] OR "biliopancreatic diversion"[All Fields] OR "biliopancreatic diversion"[MeSH Terms] OR "duodenal switch"[All Fields]) AND ("Gallstones"[All Fields] OR "Gallstones"[MeSH Terms] OR "Biliary"[All Fields] OR "Biliary Tract Diseases"[All Fields] OR "Biliary Tract Diseases"[MeSH Terms] OR "gallbladder"[All Fields] OR "gallbladder"[MeSH Terms] OR "Gallbladder diseases"[All Fields] OR "Gallbladder diseases"[MeSH Terms] OR "Cholelithiasis"[All Fields] OR "Cholelithiasis"[MeSH Terms] OR "Cholecystolithiasis"[All Fields] OR "choledocholithiasis"[All Fields] OR "Cholecystectomy"[All Fields] OR "Cholecystectomy"[MeSH Terms])) |
| --- | --- |
| Web of Science | TS=((“bariatric Surgery” OR “Bariatric” OR “Metabolic Surgery” OR “obesity surgery” OR “Gastric Bypass” OR “Roux-en-Y Gastric Bypass” OR “Gastric Band*” OR “Vertical Banded Gastroplasty” OR “Gastric Sleeve" OR “Sleeve Gastrectomy” OR “biliopancreatic diversion” OR “duodenal switch”) AND (“Gallstone” OR “Biliary” OR “Biliary Tract Diseases” OR “gallbladder” OR “Gallbladder diseases” OR “Cholelithiasis” OR “Cholecystolithiasis” OR “choledocholithiasis” OR “Cholecystectomy”)) |
| Scopus | TITLE-ABS-KEY ( "bariatric Surgery" OR "Bariatric" OR "Metabolic Surgery" OR "obesity surgery" OR "Gastric Bypass" OR "Roux-en-Y Gastric Bypass" OR "Gastric Band*" OR "Vertical Banded Gastroplasty" OR "Gastric Sleeve" OR "Sleeve Gastrectomy" OR "biliopancreatic diversion" OR "duodenal switch" ) AND TITLE-ABS-KEY ( "Gallstone" OR "Biliary" OR "Biliary Tract Diseases" OR "gallbladder" OR "Gallbladder diseases" OR "Cholelithiasis" OR "Cholecystolithiasis" OR "choledocholithiasis" OR "Cholecystectomy") |

| **Study** | **Patients** | **Postoperative BMI (Mean±SD, kg/m^2^)** | **%TWL (Mean ± SD)** | **%EWL (Mean ± SD)** | **%EBMIL (Mean ± SD)** | **Comorbidities** | | | | | |
| --- | --- | --- | --- | --- | --- | --- | --- | --- | --- | --- | --- |
|  |  |  |  |  |  | **DM (n)** | **Dyslipidemia (n)** | **HTA (n)** | **Cardiovascular Disease (n)** | **GERD (n)** | **OSA (n)** |
| Abu Abeid Subhi | 134 | 29.4 | NR | NR | NR | NR | NR | NR | NR | NR | NR |
| Aldriweesh MA | 490 | 33.2 ± 7.4 | 28 ± 12 | NR | 64 | 145 | 109 | 125 | NR | NR | 65 |
| Alimogulları M | 111 | 27.9 ± 5.5 | 37.2 ± 7.9 | 88.4 ± 18.8 | 89.3 ± 18.5 | 31 | 54 | 29 | NR | NR | 31 |
| Alsaif FA | 711 | 30.2 ± 6.5 | 32.5 ± 10.2 | NR | NR | 130 | 67 | 135 | NR | NR | NR |
| Amstutz S | 64 | 29 | NR | NR | NR | NR | NR | NR | NR | NR | NR |
| Anveden A | 1,755 | NR | 16 | NR | 60 | 296 | NR | 1377 | NR | NR | NR |
| Aridi HD | 319 | 29.4 ± 6.4 | 32.5 ± 13 | 79.5 ± 30.7 | NR | 64 | 76 | 75 | NR | NR | 60 |
| Bastouly M | 20 | 41.6 ± 5.1 | 12.4 ± 2 | 10.1 ± 2.1 | NR | NR | NR | NR | NR | NR | NR |
| Brockmeyer JR | 1,527 | NR | NR | NR | 66.1 | NR | NR | NR | NR | NR | NR |
| Chen JH | 2,317 | NR | NR | NR | NR | 149 | 11 | 220 | NR | NR | NR |
| Coupaye M | 160 | NR | 24.63 | 58.8 ± 18.2 | NR | 26 | 19 | 46 | NR | NR | NR |
| de Oliveira CLB | 103 | 34.7 | 65.53 | 68.4 | NR | NR | NR | NR | NR | NR | NR |
| Dhondt M | 625 | NR | NR | 51.3 | NR | 83 | 163 | NR | 26 | NR | NR |
| ElHadidi A | 850 | NR | NR | 65.1 ± 2.8 | NR | NR | NR | NR | NR | NR | NR |
| Guzman HM | 176 | 27.9 | NR | NR | 97.1 | 15 | 89 | 46 | NR | NR | NR |
| Hasan MY | 102 | 31.0 ± 5.7 | NR | NR | 10.6 ± 4.5 | 26 | 35 | NR | NR | NR | NR |
| Karadeniz M | 46 | 31.4 | NR | NR | NR | NR | NR | NR | NR | NR | NR |
| Kim, Jin-Jo | 454 | NR | NR | NR | NR | NR | NR | NR | NR | NR | NR |
| Kiewiet RM | 103 | 34.3 ± 6.2 | 31.5 ± 11.3 | NR | NR | NR | NR | NR | NR | NR | NR |
| Kızılkaya MC | 185 | 32.3 ± 4.0 | NR | NR | NR | NR | NR | NR | NR | NR | NR |
| Lasnibat RJP | 221 | NR | 34.55 | 83.44 | NR | NR | NR | NR | NR | NR | NR |
| Li VKM | 548 | NR | 38 ± 9.5 | NR | NR | 173 | 216 | NR | NR | NR | NR |
| Manatsathit W | 96 | 32.2 ± 7.0 | 33.5 ± 7.3 | NR | 72.1 ± 19.8 | 31 | NR | 44 | NR | NR | NR |
| Melmer A | 109 | NR | 15 | 59 | 59.8 ± 83.1 | NR | NR | NR | NR | NR | NR |
| Moon RC | 586 | 26.51 | NR | 59.4 ± 18.0 | NR | NR | NR | NR | NR | NR | NR |
| Morais M | 653 | 32.4 ± 5.4 | NR | 63.3 ± 25.7 | NR | 406 | 406 | 406 | NR | NR | NR |
| Nagem R | 38 | NR | NR | NR | 77.5 | NR | NR | NR | NR | NR | NR |
| O'Brien PE | 809 | NR | NR | 52 | NR | NR | NR | NR | NR | NR | NR |
| Ostlund M | 13,443 | NR | NR | NR | NR | NR | NR | NR | NR | NR | NR |
| Papavramidis S | 84 | 27.4 ± 8.0 | NR | 22.0 ± 8.3 | NR | NR | NR | NR | NR | NR | NR |
| Patel JA | 1,050 | NR | NR | NR | NR | NR | NR | NR | NR | NR | NR |
| Patel KR | 199 | NR | NR | NR | NR | 41 | 91 | 98 | NR | 96 | NR |
| Pineda O | 146 | NR | NR | 77.9 ± 16.1 | NR | NR | NR | NR | NR | NR | NR |
| Portenier DD | 1,057 | NR | NR | 65 | NR | NR | NR | NR | NR | NR | NR |
| Sakcak I | 137 | 36.7 ± 5.6 | NR | NR | NR | 12 | 23 | 9 | NR | 37 | 7 |
| Sioka E | 150 | NR | NR | NR | NR | NR | NR | NR | NR | NR | NR |
| Taha MIA | 103 | NR | 39.8 ± 4.1 | NR | NR | 18 | 59 | NR | NR | NR | NR |
| Tucker ON | 1,544 | NR | NR | NR | NR | NR | NR | NR | NR | NR | NR |
| Wanjura V | 33,725 | 28.26 ± 5.2 | NR | NR | NR | NR | NR | NR | NR | NR | NR |

**Supplementary Table 3.1** Basic Clinical Characteristics of included studies for risk of *de novo* post-bariatric gallstone disease and its predictive factors

| **Study** | **Symptomatic *de novo* GD** | | | | | **Subtypes of *de novo* GD** | | | | |
| --- | --- | --- | --- | --- | --- | --- | --- | --- | --- | --- |
|  | **Patients with GD (n)** | **OpenRYGBP** | **LRYGBP** | **LSG** | **LAGB** | **Biliary Colic (n)** | **Acute Cholecystitis (n)** | **Choledocholithiasis (n)** | **Cholangitis (n)** | **Acute Pancreatitis (n)** |
| Abu Abeid Subhi | 10 | 0 | 0 | 0 | 10 | NR | NR | NR | NR | NR |
| Aldriweesh MA | 32 | NR | NR | 26 | 6 | NR | NR | NR | NR | NR |
| Alimogulları M | 13 | NR | NR | 41 | NR | NR | NR | NR | NR | NR |
| Alsaif FA | 25 | NR | NR | 25 | NR | NR | NR | NR | NR | NR |
| Amstutz S | 22 | NR | 22 | NR | NR | 0 | 3 | 8 | 0 | 0 |
| Anveden A | 307 | NR | NR | NR | NR | 135 | NR | NR | NR | NR |
| Aridi HD | 24 | NR | NR | 24 | NR | NR | NR | NR | NR | NR |
| Bastouly M | 6 | 13 | NR | NR | NR | 6 | NR | NR | NR | NR |
| Brockmeyer JR | 91 | NR | NR | NR | NR | 60 | 15 | 6 | 0 | 7 |
| Chen JH | 67 | NR | NR | NR | NR | 26 | 36 | 5 | 0 | 0 |
| Coupaye M | 20 | 40 | NR | 12 | NR | 15 | 2 | 3 | 9 | 0 |
| de Oliveira CLB | 19 | 19 | NR | NR | NR | 19 | NR | NR | NR | NR |
| Dhondt M | 43 | NR | 43 | NR | NR | NR | NR | NR | NR | NR |
| ElHadidi A | 235 | 0 | 0 | 235 | 0 | 198 | 0 | 2 | 0 | 0 |
| Guzman HM | NR | 0 | 0 | 85 | 0 | NR | NR | NR | NR | NR |
| Hasan MY | 1 | 0 | 0 | 24 | 0 | 0 | 0 | 0 | 0 | 0 |
| Karadeniz M | 6 | 0 | 10 | 0 | 0 | 6 | NR | NR | NR | NR |
| Kim, Jin-Jo | 9 | NR | NR | NR | NR | NR | NR | NR | NR | NR |
| Kiewiet RM | 7 | 0 | 0 | 0 | 31 | 2 | NR | NR | NR | NR |
| Kızılkaya MC | 3 | 3 | 0 | 24 | 0 | NR | NR | NR | NR | NR |
| Lasnibat RJP | 1 | NR | NR | NR | NR | 0 | 1 | 0 | 0 | 0 |
| Li VKM | 45 | 43 | NR | 2 | NR | 35 | NR | NR | NR | NR |
| Manatsathit W | 22 | NR | NR | 48 | NR | 13 | 5 | 3 | 0 | 1 |
| Melmer A | 12 | NR | NR | 6 | 13 | NR | NR | NR | NR | NR |
| Moon RC | 28 | NR | 21 | 7 | 0 | 26 | 0 | 0 | 2 | 0 |
| Morais M | 24 | NR | NR | NR | NR | 13 | 4 | 2 | 0 | 5 |
| Nagem R | 6 | 11 | NR | NR | NR | 4 | 0 | 0 | 0 | 2 |
| O'Brien PE | 55 | NR | NR | NR | 55 | 53 | 0 | 0 | 1 | 1 |
| Ostlund M | 722 | NR | 232 | NR | NR | NR | NR | NR | NR | NR |
| Papavramidis S | 34 | NR | NR | NR | NR | 12 | 20 | 2 | 0 | 0 |
| Patel JA | 52 | NR | 52 | NR | NR | 49 | NR | NR | NR | NR |
| Patel KR | 12 | NR | 12 | NR | NR | 5 | 5 | 0 | 0 | 2 |
| Pineda O | 5 | NR | NR | NR | NR | 2 | 3 | 0 | 0 | 0 |
| Portenier DD | 80 | 80 | NR | NR | NR | 79 | 0 | 0 | 0 | 1 |
| Sakcak I | 7 | NR | NR | NR | 7 | NR | NR | NR | NR | NR |
| Sioka E | 8 | NR | NR | 8 | NR | 1 | 4 | 2 | 0 | 1 |
| Taha MIA | 22 | NR | 48 | NR | NR | NR | NR | NR | NR | NR |
| Tucker ON | 104 | NR | 104 | NR | NR | 75 | 22 | 4 | 0 | 3 |
| Wanjura V | 1133 | NR | 1133 | NR | NR | 725 | NR | NR | NR | NR |

**Supplementary Table 3.1** **(Continued)** Basic Clinical Characteristics of included studies for risk of *de novo* post-bariatric gallstone disease and its predictive factors

BMI: Body Index Mass (kg/m^2^); %TWL: percentage of total weight loss; %EWL: percentage of excess weight loss; %EBMIL: percentage of excess BMI loss; SD: Standard Deviation; DM: Diabetes Mellitus type 2; HTA: Hypertension; Cardiovascular Disease includes Ischemic heart disease (Acute coronary syndrome, Stable coronary artery disease, history of arterial revascularization), Stroke or Transient Ischemic Attack, Peripheral artery disease, Aortic aneurysm; GERD: Gastroesophageal reflux disease, OSA: Obstructive sleep apnea; OpenRYGBP: laparotomy Roux-en-Y gastric bypass; LRYGBP laparoscopic Roux-en-Y gastric bypass; LAGB laparoscopic adjustable gastric banding; LSG laparoscopic sleeve gastrectomy; GD: gallstone disease, NR: Not reported

**Supplementary Table 3.2** Studies Clinical Characteristics for risk of *de novo* symptomatic post-bariatric GD among patients with preoperative alithiasic versus lithiasic gallbladder

| **Study** | **Preoperative Alithiasic GB** | | | **Preoperative Lithiasic GB** | | |
| --- | --- | --- | --- | --- | --- | --- |
|  | **Patients at risk (n)** | **Patients with postoperative symptomatic GD (n)** | **Patients with no postoperative symptomatic GD (n)** | **Patients at risk (n)** | **Patients with postoperative symptomatic GD (n)** | **Patients with no postoperative symptomatic GD (n)** |
| D'Hondt, M. | 521 | 30 | 491 | 104 | 9 | 95 |
| El Hadidi, A. | 755 | 218 | 537 | 95 | 17 | 78 |
| Hasan, M. Y. | 87 | 1 | 86 | 15 | 0 | 14 |
| Morais, M. | 581 | 24 | 557 | 72 | 0 | 72 |
| Pineda, O. | 97 | 2 | 95 | 49 | 3 | 46 |
| Portenier, D. D. | 379 | 37 | 342 | 81 | 14 | 67 |
| Sioka, E. | 106 | 5 | 101 | 23 | 3 | 20 |
| Tucker, O. N. | 1462  GB: Gallbladder; GD: Gallstone Disease | 88 | 1374 | 82 | 16 | 66 |

**Supplementary Table 3.3** Basic Clinical Characteristics of included studies for comparison of the morbidity and mortality of bariatric surgery alone versus prophylactic cholecystectomy concomitant to bariatric surgery

TABLE S3.3 Basic Clinical Characteristics of included studies for comparison of the morbidity and mortality of bariatric surgery versus prophylactic cholecystectomy concomitant to bariatric surgery

TABLE S3.3 Basic Clinical Characteristics of included studies for comparison of the morbidity and mortality of bariatric surgery versus prophylactic cholecystectomy concomitant to bariatric surgery

TABLE S3.3 Basic Clinical Characteristics of included studies for comparison of the morbidity and mortality of bariatric surgery versus prophylactic cholecystectomy concomitant to bariatric surgery

| **Study** | **Patients (n)** | **Postoperative BMI (kg/m2)** | **%TWL (Mean ± SD)** | **%EWL (Mean ± SD)** | **Comorbidities** | | | | | |  |
| --- | --- | --- | --- | --- | --- | --- | --- | --- | --- | --- | --- |
|  |  |  |  |  | **DM (n)** | **HTA (n)** | **Cardiovascular Disease (n)** | **COPD (n)** | **OSA (n)** | **CKD (n)** |  |
|  |  |  |  |  |  |  |  |  |  |  |  |
| Ahmed AR | 400 | NR | NR | NR | NR | NR | NR | NR | NR | NR |  |
| Coskun H | 48 | NR | NR | NR | NR | NR | NR | NR | NR | NR |  |
| Dakour-Aridi HN | 21,137 | NR | NR | NR | 4,826 | 10,382 | 40 | 269 | 0 | 0 |  |
| Dorman RB | 32,946 | NR | NR | NR | 10,058 | 18,339 | 36 | 617 | 0 | 0 |  |
| Juo, Yen-Yi | 553,659 | NR | NR | NR | NR | NR | NR | NR | 0 | 0 |  |
| Kim, Jin-Jo | 752 | NR | NR | NR | NR | NR | NR | NR | 0 | 0 |  |
| Nougou A | 772 | NR | NR | NR | NR | NR | NR | NR | 0 | 0 |  |
| Santos BF | 33,075 | NR | NR | NR | NR | NR | NR | NR | 0 | 0 |  |
| Sucandy I | 361 | NR | NR | 82.2 ± 45.5 | 115 | 133 | 12 | 0 | 145 | 0 |  |
| Tarantino I | 274 | NR | NR | 77.3 ± 26.8 | NR | NR | NR | NR | NR | NR |  |
| Tucker ON | 1,669 | NR | NR | NR | NR | NR | NR | NR | NR | NR |  |
| Wanjura V | 33,725 | 28.3 ± 5.2 | NR | NR | NR | NR | NR | NR | NR | NR |  |
| Wood SG | 4,048 | NR | NR | NR | 820 | 1,929 | NR | 0 | 0 | 14 |  |
| Wood SG | 2,820 | NR | NR | NR | 929 | 1,431 | NR | 0 | 0 | 23 |  |
| Zilberstein B | 308 | NR | NR | NR | NR | NR | NR | NR | NR | NR |  |

BMI: Body Index Mass (kg/m^2^); %TWL: percentage of total weight loss; %EWL: percentage of excess weight loss; SD: Standard Deviation; DM: Diabetes Mellitus type 2; HTA: Hypertension; Cardiovascular Disease includes Ischemic heart disease (Acute coronary syndrome, Stable coronary artery disease, history of arterial revascularization), Stroke or Transient Ischemic Attack, Peripheral artery disease, Aortic aneurysm; COPD: Chronic obstructive pulmonary disease, OSA: Obstructive sleep apnea, CKD: Chronic kidney disease; NR: Not reported

**Supplementary Table 3.4** Specific major postoperative complications of included studies for comparison of the morbidity and mortality of bariatric surgery alone versus prophylactic cholecystectomy concomitant to bariatric surgery

| **Study** | **Patients (n)** | **Postoperative Complications** | | | | | | | | | | | | | | | |
| --- | --- | --- | --- | --- | --- | --- | --- | --- | --- | --- | --- | --- | --- | --- | --- | --- | --- |
|  |  | **CA requiring CPR (n)** | | **Sepsis (n)** | | **Pneumonia (n)** | | **AKD (n)** | | **TVE (n)** | | **Bleeding requiring transfusion (n)** | | **Organ-space surgical site infection (n)** | | **Anastomosis leakage (n)** | |
|  |  | **BS + CCY** | **BS Alone** | **BS + CCY** | **BS Alone** | **BS + CCY** | **BS Alone** | **BS + CCY** | **BS Alone** | **BS + CCY** | **BS Alone** | **BS + CCY** | **BS Alone** | **BS + CCY** | **BS Alone** | **BS + CCY** | **BS Alone** |
| Ahmed AR | 400 | NR | NR | 3 | 1 | NR | NR | NR | NR | NR | NR | 1 | 3 | NR | NR | NR | NR |
| Coskun H | 48 | NR | NR | NR | NR | NR | NR | NR | NR | NR | NR | NR | NR | NR | NR | NR | NR |
| Dakour-Aridi HN | 21 137 | 0 | 10 | 2 | 94 | 4 | 60 | 0 | 15 | NR | NR | 8 | 193 | 2 | 102 | NR | NR |
| Dorman RB | 32 946 | 4 | 40 | 35 | 353 | 23 | 217 | 8 | 122 | 2 | 72 | 10 | 102 | 23 | 241 | NR | NR |
| Juo, Yen-Yi | 553 659 | NR | NR | NR | NR | NR | NR | NR | NR | NR | NR | NR | NR | NR | NR | NR | NR |
| Kim, Jin-Jo | 752 | 0 | 0 | NR | NR | 2 | 9 | NR | NR | 1 | 2 | 1 | 4 | NR | NR | 5 | 8 |
| Nougou A | 772 | NR | NR | NR | NR | NR | NR | NR | NR | NR | NR | NR | NR | NR | NR | NR | NR |
| Santos BF | 33 075 | NR | NR | NR | NR | NR | NR | NR | NR | NR | NR | NR | NR | NR | NR | NR | NR |
| Sucandy I | 361 | NR | NR | NR | NR | NR | NR | NR | NR | 0 | 8 | 0 | 4 | NR | NR | 0 | 1 |
| Tarantino I | 274 | NR | NR | NR | NR | NR | NR | NR | NR | NR | NR | NR | NR | NR | NR | NR | NR |
| Tucker ON | 1 669 | NR | NR | NR | NR | NR | NR | NR | NR | NR | NR | NR | NR | NR | NR | 1 | NR |
| Wanjura V | 33 725 | NR | NR | NR | NR | NR | NR | NR | NR | NR | NR | NR | NR | NR | NR | NR | NR |
| Wood SG | 4 048 | 4 | 0 | 5 | 2 | 4 | 4 | 1 | 0 | 2 | 3 | 10 | 14 | 10 | 4 | 0 | 0 |
| Wood SG | 2 820 | 1 | 1 | 7 | 28 | 8 | 5 | 4 | 3 | 1 | 3 | 19 | 11 | 2 | 7 | NR | NR |
| Zilberstein B | 308 | NR | NR | NR | NR | NR | NR | NR | NR | NR | NR | NR | NR | 0 | 14 | NR | NR |

|  |  |  |  |  |  |
| --- | --- | --- | --- | --- | --- |
| **Conversion rate of laparoscopic surgery (n)** | | **Reoperation (n)** | | **Readmission within 30 days (n)** | |
| **BS + CCY** | **BS Alone** | **BS + CCY** | **BS Alone** | **BS + CCY** | **BS Alone** |
| NR | NR | NR | NR | NR | NR |
| NR | NR | NR | NR | NR | NR |
| NR | NR | NR | NR | NR | NR |
| NR | NR | 71 | 899 | NR | NR |
| NR | NR | NR | NR | 53 | 1 071 |
| NR | NR | NR | NR | NR | NR |
| NR | NR | NR | NR | NR | NR |
| NR | NR | NR | NR | NR | NR |
| 1 | 4 | NR | NR | NR | NR |
| 5 | 4 | NR | NR | NR | NR |
| 1 | NR | NR | NR | NR | NR |
| NR | NR | 5 | 835 | NR | NR |
| 3 | 0 | 33 | 14 | 83 | 62 |
| 1 | 2 | 38 | 32 | 77 | 83 |
| NR | NR | NR | NR | NR | NR |

**Supplementary Table 3.4 (Continued)** Specific major postoperative complications of included studies for comparison of the morbidity and mortality of bariatric surgery alone versus prophylactic cholecystectomy concomitant to bariatric surgery

CA requiring CPR: Cardiac Arrest requiring cardiopulmonary resuscitation, TVE: venous thromboembolism, AKD: Acute kidney disease, BS: bariatric surgery; BS+CCY: prophylactic cholecystectomy concomitant to bariatric surgery; NR: not reported

|  | **Number of studies** | **Number of patients** | **Univariable metaregression—OR (95% CrI)  [% iterations with OR < 1]** |
| --- | --- | --- | --- |
|  |  |  |  |
| Year of publication | 23 | 38,210 | 1.00 (0.99-1.00) [>99%] |
| Study Design |  |  |  |
| Prospective design | 6 | 1,746 | b |
| Retrospective design | 17 | 36,464 | 0.92 (0.22-2.53) [70%] |
| Mean Age | 15 | 36,505 | 1.05 (0.87-1.17) [32%] |
| Proportion of females patients | 17 | 36,672 | 1.02 (0.98-1.06) [23%] |
| Preoperative average of BMI | 16 | 36,575 | 1.11 (0.99-1.22) [4%] |
| Follow-up | 17 | 36,672 | 1.00 (0.95-1.05) [43%] |
| Preoperative Gallbladder Status |  |  |  |
| Lithiasic GB | 7 | 36,034 | b |
| Alithiasic GB | 16 | 2,176 | 2.57 (0.50-7.93) [14%] |
| Type of BS |  |  |  |
| No RYGBP subgroup | 14 | 37,385 | b |
| RYGBP subgroup | 8 | 728 | 1.59 (0.35-4.47) [31%] |
| No LRYGBP subgroup | 17 | 2,896 | b |
| LRYGBP subgroup | 6 | 35,314 | 0.62 (0.17-1.56) [85%] |
| No LSG subgroup | 14 | 36,693 | b |
| LSG subgroup | 9 | 1,517 | 1.66 (0.52-4.47) [25%] |
| No LAGB subgroup | 17 | 37,721 | b |
| LAGB subgroup | 6 | 489 | 0.68 (0.16-1.83) [82%] |
| Quality rating |  |  |  |
| Poor/Fair quality | 9 | 35,093 | b |
| High quality | 14 | 3,117 | 1.09 (0.49-2.30) [49%] |

**Supplementary Table S4**. Results of metaregression for risk of *de novo* post-bariatric asymptomatic or symptomatic gallstone disease

BMI: body mass index (kg/m2); OpenRYGBP: laparotomy Roux-en-Y gastric bypass; LRYGBP: laparoscopic Roux-en-Y gastric bypass; LAGB: laparoscopic adjustable gastric banding; LSG: laparoscopic sleeve gastrectomy; GB: Gallbladder; OR: odds ratio; CrI: Credible Intervals

^b^ Reference category

**Supplementary Table 5.1** Detailed results of the risk of bias assessments for included primary studies regarding the risk of *de novo* post-bariatric gallstone disease and its predictive factors

| **Studies** | **Q1** | **Q2** | **Q3** | **Q4** | **Q5** | **Q6** | **Q7** | **Q8** | **Q9** | **Q10** | **Q11** | **Q12** | **Q13** | **Q14** | **Quality Rating** |
| --- | --- | --- | --- | --- | --- | --- | --- | --- | --- | --- | --- | --- | --- | --- | --- |
| Abu Abeid Subhi | Y | Y | Y | Y | N | Y | Y | N | N | N | Y | NA | NR | N | FAIR |
| Aldriweesh, M. A. | Y | Y | Y | Y | N | Y | Y | Y | Y | Y | Y | NA | Y | N | GOOD |
| Alimoğulları, M. | Y | Y | N | Y | N | Y | Y | N | Y | Y | Y | NA | Y | Y | GOOD |
| Alsaif, F. A. | Y | Y | Y | Y | N | Y | Y | N | Y | Y | Y | NA | Y | N | GOOD |
| Amstutz, S. | Y | Y | Y | Y | N | Y | Y | N | Y | N | Y | NA | Y | N | FAIR |
| Anveden, Å | Y | Y | Y | Y | N | Y | Y | Y | Y | Y | Y | NA | Y | Y | GOOD |
| Aridi, H. D | Y | Y | Y | Y | Y | Y | Y | Y | Y | Y | Y | NA | Y | Y | GOOD |
| Bastouly, Michel | Y | Y | CD | Y | N | Y | Y | N | Y | N | Y | NA | NR | N | POOR |
| Brockmeyer, J. R. | Y | Y | N | Y | N | Y | Y | N | N | N | Y | NA | Y | N | FAIR |
| Chen, J. H. | Y | Y | N | Y | N | Y | Y | N | Y | N | Y | NA | Y | Y | GOOD |
| Coupaye, M. | Y | Y | Y | Y | N | Y | Y | Y | Y | Y | Y | NA | Y | N | GOOD |
| de Oliveira, C. L. B. | Y | Y | Y | Y | N | Y | Y | N | Y | N | Y | NA | NR | N | FAIR |
| D'Hondt, M. | Y | Y | Y | Y | N | Y | Y | Y | Y | Y | Y | NA | Y | Y | GOOD |
| El Hadidi, A. | Y | Y | Y | Y | N | Y | Y | N | Y | Y | Y | NA | Y | Y | GOOD |
| Guzmán, H. M. | Y | Y | Y | Y | N | Y | Y | N | Y | N | Y | NA | Y | N | FAIR |
| Hasan, M. Y. | Y | Y | NA | Y | N | Y | Y | Y | Y | N | Y | NA | Y | N | FAIR |
| Karadeniz, Metin | Y | Y | Y | Y | N | Y | Y | N | Y | N | Y | NA | NR | N | FAIR |
| Kim, Jin-Jo | Y | Y | Y | Y | N | Y | Y | Y | Y | N | Y | NA | Y | N | GOOD |
| Kiewiet, R. M. | Y | Y | Y | Y | N | Y | Y | Y | Y | N | Y | NA | NR | N | GOOD |
| Kızılkaya, M. C. | Y | Y | Y | Y | N | Y | Y | N | Y | N | Y | NA | Y | N | GOOD |
| Lasnibat R, J. P. | Y | Y | Y | Y | N | Y | Y | N | Y | N | Y | NA | N | N | FAIR |
| Li, V. K. M. | Y | Y | Y | Y | N | Y | Y | Y | Y | N | Y | NA | Y | N | FAIR |
| Manatsathit, W. | Y | Y | N | Y | N | Y | Y | Y | Y | Y | Y | NA | NR | N | GOOD |
| Melmer, A. | Y | Y | Y | Y | N | Y | Y | Y | Y | N | Y | NA | Y | N | GOOD |
| Moon, R. C. | Y | Y | Y | Y | N | Y | Y | Y | Y | Y | Y | NA | Y | N | GOOD |
| Morais, M. | Y | Y | Y | Y | N | Y | Y | Y | Y | Y | Y | NA | Y | Y | GOOD |
| Nagem, R. | Y | Y | CD | Y | N | Y | Y | Y | Y | Y | Y | NA | NR | N | GOOD |
| O'Brien, P. E. | Y | Y | Y | Y | N | Y | Y | N | N | N | Y | NA | NR | N | FAIR |
| Ostlund M. | Y | Y | CD | Y | N | Y | Y | Y | Y | Y | Y | NA | NR | N | GOOD |
| Papavramidis, S. | Y | Y | Y | Y | N | Y | Y | N | Y | Y | Y | NA | NR | N | FAIR |
| Patel, J. A. | Y | Y | Y | Y | N | Y | Y | N | Y | Y | Y | NA | Y | N | FAIR |
| Patel, K. R. | Y | Y | N | Y | N | Y | Y | Y | Y | Y | Y | NA | Y | N | GOOD |
| Pineda, O. | Y | Y | Y | Y | N | Y | Y | N | Y | Y | Y | NA | Y | N | GOOD |
| Portenier, D. D. | Y | Y | Y | Y | N | Y | Y | Y | Y | CD | Y | NA | NR | N | FAIR |
| Sakcak, I. | Y | Y | Y | Y | N | Y | Y | Y | Y | N | Y | NA | Y | N | FAIR |
| Sioka, E. | Y | Y | Y | Y | N | Y | Y | Y | Y | Y | Y | NA | Y | N | GOOD |
| Taha, M. I. A. | Y | Y | Y | Y | N | Y | Y | Y | Y | Y | Y | NA | Y | N | GOOD |
| Tucker, O. N. | Y | Y | N | Y | N | Y | Y | N | Y | N | Y | NA | NR | N | FAIR |
| Wanjura, V. | Y | N | Y | Y | Y | Y | Y | Y | Y | Y | N | NA | Y | Y | FAIR |

Q: Question; Y: yes; N: No; CD, cannot determine; NA, non-applicable; NR, not reported

| **Studies** | **Q1**  **Supplementary Table 5.2** Detailed results of the risk of bias assessments for included primary studies regarding the comparison of the morbidity and mortality of bariatric surgery alone versus prophylactic cholecystectomy concomitant to bariatric surgery | **Q2** | **Q3** | **Q4** | **Q5** | **Q6** | **Q7** | **Q8** | **Q9** | **Q10** | **Q11** | **Q12** | **Q13** | **Q14** | **Quality Rating** |
| --- | --- | --- | --- | --- | --- | --- | --- | --- | --- | --- | --- | --- | --- | --- | --- |
| Ahmed, A. R. | Y | Y | Y | Y | N | Y | Y | N | Y | N | Y | NA | NR | N | GOOD |
| Coskun, H. | Y | Y | Y | Y | N | Y | Y | N | Y | N | Y | NA | Y | NR | FAIR |
| Dakour-Aridi, H. N. | Y | Y | Y | Y | Y | Y | Y | Y | Y | N | Y | NA | NR | Y | GOOD |
| Dorman, R. B | Y | Y | N | Y | N | Y | Y | Y | Y | Y | Y | NA | Y | Y | GOOD |
| Juo, Yen-Yi | Y | Y | Y | Y | N | Y | Y | Y | Y | Y | Y | NA | Y | Y | GOOD |
| Kim, Jin-Jo | Y | Y | Y | Y | N | Y | Y | Y | Y | Y | Y | NA | Y | N | GOOD |
| Nougou, A. | Y | Y | Y | Y | N | Y | Y | N | Y | N | Y | NA | NR | N | FAIR |
| Santos, B. F. | Y | Y | Y | Y | N | Y | Y | NR | Y | NR | Y | NA | NR | NR | FAIR |
| Sucandy, Iswanto | Y | Y | Y | Y | N | Y | Y | Y | Y | Y | Y | NA | N | N | FAIR |
| Tarantino, I. | Y | Y | Y | Y | N | Y | Y | Y | Y | Y | Y | NA | N | Y | GOOD |
| Tucker, O. N. | Y | Y | N | Y | N | Y | Y | N | Y | N | Y | NA | NR | N | FAIR |
| Wanjura, V. | Y | N | Y | Y | Y | Y | Y | Y | Y | Y | N | NA | Y | Y | FAIR |
| Wood, S. G. | Y | Y | Y | Y | N | Y | Y | Y | Y | Y | Y | NA | N | Y | GOOD |
| Zilberstein, B. | Y | Y | Y | Y | N | Y | Y | N | Y | N | Y | NA | NR | N | FAIR |

Q: Question; Y: yes; N: No; CD: cannot determine; NA: non-applicable; NR: not reported

Risk of bias assessments are based on National Institutes of Health quality (NIH) assessment criteria for observational studies - this tool consists of a form with 14 Y(yes) or N (no) questions (related to the research question, study population, exposure, outcome, blinding, follow-up, and statistical analyses) and a final quality rating – G (good), F (fair), P (poor), classifying the study according to its potential risk of bias.

**PRISMA checklist**

| **Section/topic** | **#** | **Checklist item** | **Reported on page and paragraph/ table #** |
| --- | --- | --- | --- |
| **TITLE** |  | | |
| Title | 1 | Identify the report as a systematic review, meta-analysis, or both. | Title Page - Page 1 & 2  “Risk and Prophylactic Management of Gallstone Disease in Bariatric Surgery: A Systematic Review and A Bayesian meta-analysis” |
| **ABSTRACT** |  | | |
| Structured summary | 2 | Provide a structured summary including, as applicable: background; objectives; data sources; study eligibility criteria, participants, and interventions; study appraisal and synthesis methods; results; limitations; conclusions and implications of key findings; systematic review registration number. | Page 3 & 4 |
| **INTRODUCTION** |  | | |
| Rationale | 3 | Describe the rationale for the review in the context of what is already known | Page 5-6 (Paragraph 1-3) “Despite its benefits, BS is associated with a 3-28 % incidence of symptomatic gallstone disease (GD) (…)”; “Understanding the risk factors associated with the development of GD may be crucial for risk stratification and distinct patient management (…)”; “The varying incidence of symptomatic GD after BS has resulted in controversies regarding whether prophylactic concomitant cholecystectomy (CCY) should be performed. Currently, there are three approaches on this subject (…)”; “There may be some arguments in favor or against for each of these options (…)” |
| Objectives | 4 | Provide an explicit statement of questions being addressed with reference to participants, interventions, comparisons, outcomes, and study design (PICOS) | Page 5-6 (Paragraph 4) “In this systematic review and meta-analysis, we aimed to (…)” |
| **METHODS** |  | | |
| Protocol and registration | 5 | Indicate if a review protocol exists, if and where it can be accessed (e.g., Web address), and, if available, provide registration information including registration number. | Not registered |
| Eligibility criteria | 6 | Specify study characteristics (e.g., PICOS, length of follow-up) and report characteristics (e.g., years considered, language, publication status) used as criteria for eligibility, giving rationale. | Page 7 (Paragraph 2-4) (Supplementary Table 1) “We included observational studies assessing BS as an obesity treatment for patients with a BMI ≥ 40 kg/m2 or BMI ≥ 35 kg/m2 with weight-related comorbidities”; “For objectives i and ii (risk of de novo post-bariatric GD and its predictive factors), the outcome to be reported was GD development”; “For objective iii (comparison of the morbidity and mortality of BS alone versus BS + prophylactic CCY), any of the following outcomes needed to be reported: postoperative mortality, duration of surgery, hospital length-of-stay (LOS), and major postoperative complications.”; “More detailed inclusion and exclusion criteria, for each specific objective, are shown in Supplementary Table 1.”; “No restrictions were set for language or publication year.” |
| Information sources | 7 | Describe all information sources (e.g., databases with dates of coverage, contact with study authors to identify additional studies) in the search and date last searched. | Page 7-8 (Paragraph 4) “We searched three electronic databases (PubMed, EMBASE, and Web of Science) through April 2021.” “This search was supplemented by gray literature search (conference papers, clinical trials – ongoing or unpublished), as well as hand-searching references of included primary studies and other relevant reviews” |
| Search | 8 | Present full electronic search strategy for at least one database, including any limits used, such that it could be repeated. | Page 7 (Paragraph 4; Supplementary Table 2) “Search queries are detailed in Supplementary Table 2.” |
| Study selection | 9 | State the process for selecting studies (i.e., screening, eligibility, included in systematic review, and, if applicable, included in the meta-analysis). | Page 8-9 (Paragraph 5,7) (Figure 1) “After removing duplicates, each study was independently assessed by two reviewers (F.C and M.R), first by title and abstract screening, and then by full-text reading.” “In study selection or data extraction, any disagreements between reviewers were resolved by consulting a third senior reviewer (H.S.S) to reach a final decision.” |
| Data collection process | 10 | Describe method of data extraction from reports (e.g., piloted forms, independently, in duplicate) and any processes for obtaining and confirming data from investigators. | Page 8 (Paragraph 5); Page 9 (Paragraph 6 & 7) “(…) independently assessed by two reviewers (F.C and M.R) (…)”; “Two reviewers independently extracted data of selected studies using a predefined form purposely built for this study.”; “Whenever provided, we retrieved data separately based on preoperative gallbladder status. Data related to other biliary conditions, such as gallbladder carcinoma or polyps, were not retrieved.”; “If distinct eligible publications reported data on the same patient cohort, the more recent and largest cohort was included. Authors were contacted whenever full texts were not available or to provide the relevant missing information. In study selection or data extraction, any disagreements between reviewers were resolved by consulting a third senior reviewer (H.S.S) to reach a final decision.” |
| Data items | 11 | List and define all variables for which data were sought (e.g., PICOS, funding sources) and any assumptions and simplifications made. | Page 8-9 (Paragraph 6 & 7); “For each primary study, the following information was retrieved: (…)”; “An alithiasic gallbladder was defined as a preoperative gallbladder without gallstones or sludge, and a lithiasic gallbladder was defined as a preoperative asymptomatic gallbladder with gallstones or sludge without being submitted to CCY.”; “For objectives i and ii (risk of de novo post-bariatric GD and its predictive factors), we also retrieved information on the number of patients: (…)”; “Both patients with no symptoms of cholelithiasis and, either preoperative negative gallstone findings or preoperative positive gallstone findings, were at risk of de novo symptomatic GD. In contrast, only patients with preoperative negative gallstone findings and primarily asymptomatic were considered at risk for de novo asymptomatic GD.”; “For objective iii (comparison between BS alone versus BS + prophylactic CCY ), the additional following information was concerned (…)” |
| Risk of bias in individual studies / Risk of bias across studies | 12/  15 | Describe methods used for assessing risk of bias of individual studies (including specification of whether this was done at the study or outcome level), and how this information is to be used in any data synthesis. | Page 9 (Paragraph 8) “The quality of primary studies was independently assessed by two researchers (F.C and M.R) using the National Institutes of Health quality assessment criteria for observational studies. To reach a consensus, divergent opinions about quality assessment were discussed with a third reviewer (H.S.S). This tool consists of a form with 14 yes-or-no questions (…) and a final quality rating (good, fair, or poor), classifying the study according to its potential risk of bias (…)” |
| Summary measures | 13 | State the principal summary measures (e.g., risk ratio, difference in means). | Page 10 (Paragraph 10) “In this study, for the risk of de novo post-bariatric GD, we computed the meta-analytical risk of GD and of symptomatic GD only. To compare outcomes between patients submitted to BS alone as index events versus those submitted to BS + prophylactic CCY, we computed meta-analytical odds ratio (OR) or mean differences (MD) depending on whether outcome variables were categorical or continuous, respectively”. |
| Synthesis of results | 14 | Describe the methods of handling data and combining results of studies, if done, including measures of consistency (e.g., I^2^) for each meta-analysis. | Page 10 (Paragraph 10 & 12) “Of these results, we collected information on the mean values and respective 95% credible intervals (…)”; “Heterogeneity was assessed through an estimate of the *I^2^* statistic – an *I*^2^>50% indicated substantial heterogeneity.” |
| Additional analyses | 16 | Describe methods of additional analyses (e.g., sensitivity or subgroup analyses, meta-regression), if done, indicating which were pre-specified. | Page 10-11 (Paragraph 12) “Heterogeneity sources were explored through univariable meta-regression and subgroup analyses – in particular, meta-regression allowed for the identification of potential predictive factors for the risk of de novo bariatric GD. Exponentials of the meta-regression coefficients were interpreted as OR. Finally, we also performed a separate meta-analysis for the development of symptomatic GD among patients with preoperative lithiasic versus alithiasic gallbladder.” |
| **RESULTS** |  | | |
| Study selection | 17 | Give numbers of studies screened, assessed for eligibility, and included in the review, with reasons for exclusions at each stage, ideally with a flow diagram. | Page 12 (Paragraph 1) (Figure 1) “The electronic literature search resulted in 5 082 articles, of which 1 808 were duplicates. After excluding 3 184 records in the screening phase, 90 articles were fully read, of which a total of 42 were included in the systematic review (Figure 1). Hand-searching resulted in 23 additional articles, of which 8 were included.”; “In total, 50 articles were included (…)” |
| Study characteristics | 18 | For each study, present characteristics for which data were extracted (e.g., study size, PICOS, follow-up period) and provide the citations. | Page 12-13 (Paragraph 2-4) “A summary of included studies is presented in Table 1 and Table 2.1 - 2.2. The remaining characteristics are reported in Supplementary Table 3.1 - 3.4.” |
| Risk of bias within and across studies | 19/ 22 | Present data on risk of bias of each study and, if available, any outcome level assessment (see item 12). | Page 15 (Paragraph 11-12) (Table 1) (Supplementary Table 5.1 - 5.2) “The results of the risk of bias assessments for included primary studies are presented in Table 1 and a detailed description is reported in Supplementary Table 5.1 - 5.2. Most studies (n=47, 94%) did not justify sample size, and 39 studies (78%) did not adjust for any potential confounding variables. For the remaining parameters, most studies were associated with a low risk of bias.” |
| Results of individual studies | 20 | For all outcomes considered (benefits or harms), present, for each study: (a) simple summary data for each intervention group (b) effect estimates and confidence intervals, ideally with a forest plot. | Point a) Page 13 (Paragraph 5-6) (Supplementary Table 3.1) “A total of 38 210 asymptomatic patients with only preoperative negative GD findings were at risk of de novo postoperative GD.”; “A total of 63 938 asymptomatic patients with either preoperative negative or positive gallstone findings were at risk of de novo symptomatic GD. A total of 3 312 developed symptomatic GD (…)The three most common clinical presentations were biliary colic (n=1 559, 65.5%), cholecystitis (n=120, 14.7%) and symptomatic choledocholithiasis (n=37, 4.5%) (Supplementary Table 3.1). Almost all patients required postoperative cholecystectomy (n= 3 179, 96%)  Page 13-14 (Paragraph 8 & 10) (Table 3.2) (Supplementary Table 3.4)  Point b) since a Bayesian meta-analysis was performed, we computed effect measures (odds ratio (OR) or mean differences (MD) depending on whether outcome variables were categorical or continuous) and respective 95% credible intervals. (instead of confidence intervals).  Page 13 (Paragraph 5 & 7) Page 14 (Paragraph 9) (Supplementary Table 4, Table 3.1, Table 3.3) “Other results of univariable meta-regression are presented in Supplementary Table 4.”; “The results of univariable meta-regression and subgroup analyses are presented in Table 3.1”; “Univariable meta-regression results are presented in Table 3.3.”; |
| Synthesis of results | 21 | Present results of each meta-analysis done, including confidence intervals and measures of consistency. | Page 13-15 (Paragraph 5, 6, 8 & 10) (Table 3.1, Table 3.2) “The Bayesian meta‐analysis identified a post-bariatric risk of de novo GD of 20.7% (95% CrI = 13.0-29.7%), even though with severe heterogeneity (*I*^2^=75.4%)”; “corresponding to a meta-analytical risk of 8.2% (95% CrI = 5.9-11.1%), even though with high heterogeneity (*I*^2^=66.9%, Table 3.1)”; “Bayesian meta‐analysis identified that postoperative mortality was not substantially different between BS alone versus BS + prophylactic CCY (OR=0.79; 95%CrI=0.03-3.02; *I*^2^=20.7%). BS + prophylactic CCY was associated with 97% probability of higher number of postoperative major complications compared to BS alone (OR=1.74, 95%CrI=0.97-3.55; *I*^2^=56.5%).”; “Patients submitted to BS + prophylactic CCY had a longer operative time - more 29.2 minutes in the operating room (95%CrI=17.9-40.7), even though there was severe heterogeneity found (*I*^2^=89.3%). There were no relevant differences in hospital LOS (MD=-0.1 days; 95%CrI=-1.0-0.5; *I*^2^=74.3%) (Table 3.2).” |
| Additional analysis | 23 | Give results of additional analyses, if done (e.g., sensitivity or subgroup analyses, meta-regression [see Item 16]). | Page 13 (Paragraph 5) (Supplementary Table 4) “A higher BMI was associated – with a 94% probability – with higher odds of de novo GD (OR 1.11; 95%CrI = 0.99-1.22)”  Page 13 (Paragraph 7-8) (Table 3.1) “Regarding studies’ methodological characteristics, retrospective studies were associated – with a 94% probability - with lower odds of de novo symptomatic GD (OR=0.60; 95% CrI = 0.31-1.10). Pre-operative average BMI (OR=1.04; 95%CrI=0.92-1.17) and female patients’ proportion (OR=1.00; 95%CrI=0.98-1.04) did not impact the risk of de novo symptomatic GD.” “(…) laparoscopic gastric banding (LAGB) was associated – with an 82% probability - with lower odds of de novo symptomatic GD. Insufficient data on co-morbidities and weight loss after surgery did not allow meta-regression analysis models to include these variables.”;  Page 14 (Paragraph 10) (Table 3.3) “Neither age (OR=1.22, 95%CrI=0.87-1.68) nor female patients’ proportion (OR=0.95, 95%CrI=0.87-1.04) associated with a relevant impact on the association between BS alone versus BS + prophylactic CCY on the occurrence of postoperative major complications. Among bariatric surgery types, laparoscopic gastric banding (LAGB) had the greatest probability (87%) associating with a weaker association between BS alone versus BS + prophylactic CCY postoperative major complications (OR=0.42, 95%CrI=0.04-1.97).” |
| **DISCUSSION** |  | | |
| Summary of evidence | 24 | Summarize the main findings including the strength of evidence for each main outcome; consider their relevance to key groups (e.g., healthcare providers, users, and policy makers). | Page 16 (Paragraph 1) “The main findings of our study were the following: (1) the risk of developing de novo symptomatic gallstone disease after BS is not substantially high (8.2%), although three times higher than healthy population, (2) GD predictive factors after BS are not similar to those of general population, except preoperative average of BMI in asymptomatic or symptomatic GD; (3) patients who underwent prophylactic CCY had a longer operative time and a higher rate of postoperative complications than those who underwent BS alone, but mortality and hospital LOS were similar.”  Regarding strength of evidence, (i) study design was mentioned along discussion - Page 16 (Paragraph 2) “Our risk might be slightly underestimated since a clear decrease in the risk of de novo symptomatic GD was observed for retrospective studies, which might be explained by information bias. In fact, considering only prospective studies, this risk rises to 11.2%.”; Page 19 (Paragraph 8) “First, severe heterogeneity was found, explained by different study designs (…)”; (ii) study quality was mentioned also along discussion – Page 19 (Paragraph 8) “Third, almost half of the included studies did not have a low risk of bias, which could impact our results on the risk of de novo GD and postoperative complications rate, particularly, as primary studies’ quality was found to be a moderator variable of heterogeneity.” |
| Limitations | 25 | Discuss limitations at study and outcome level (e.g., risk of bias), and at review-level (e.g., incomplete retrieval of identified research, reporting bias). | Page 19 (Paragraph 8) “This systematic review has some limitations worth noting (…)” |
| Conclusions | 26 | Provide a general interpretation of the results in the context of other evidence, and implications for future research. | Page 20 (Paragraph 10) “In conclusion, after BS, the risk of developing GD is not substantially high, and severe biliary complications are extremely rare”; “Although there were no substantial differences in postoperative mortality or hospital length-of-stay, the risk of symptomatic GD and the higher risk of postoperative complications do not seem to justify performing prophylactic CCY in patients with alithiasic gallbladder. Doubts remain if a selective approach is advantageous since patients with preoperative gallbladder pathology have some increased risk of symptomatic GD.” |
| **FUNDING** |  | | |
| Funding | 27 | Describe sources of funding for the systematic review and other support (e.g., supply of data); role of funders for the systematic review. | Page 21 (Paragraph 4) “The authors declare that they have no conflict of interest.” |

*From:*  Moher D, Liberati A, Tetzlaff J, Altman DG, The PRISMA Group (2009). Preferred Reporting Items for Systematic Reviews and Meta-Analyses: The PRISMA Statement. PLoS Med 6(7): e1000097. doi:10.1371/journal.pmed1000097

For more information, visit: **www.prisma-statement.org**.
